# TGM2-P2RX7 loop promotes gemcitabine resistance in pancreatic cancer by modulating glutamine metabolism and mitophagy

**DOI:** 10.1038/s41420-025-02922-x

**Published:** 2025-12-30

**Authors:** Ke Ye, Shuhua Zhou, Xuejun Gong, Zhongcheng Zhu, Moyan Xiao, Shuai Liang

**Affiliations:** 1 Department of Hepatology, Xiangya Hospital, Central South University, Changsha, China; 2 Comprehensive Surgery, Xiangya Boai Rehabilitation Hospital, Changsha, Hunan China; 3 Department of Pancreatic Surgery, Xiangya Hospital, Central South University, Changsha, China

**Keywords:** Tumour biomarkers, Cancer metabolism

## Abstract

Pancreatic ductal adenocarcinoma (PDAC) is a highly lethal type of cancer with poor diagnosis and prognosis, and overcoming gemcitabine-resistant (Gem-R) is a major obstacle in its treatment. Given the important role of glutamine (Glu) metabolism in tumor drug resistance, we investigated the role and exact mechanism of transglutaminase type 2 (TGM2) in influencing PDAC sensitivity to gemcitabine. In this study, we found that TGM2 exhibited elevated expression levels in Gem-R cells and tissue samples from patients with clinically resistant PDAC. Mechanistically, downregulation of TGM2 suppressed the proliferation of Gem-R PDAC cells both in vitro and in vivo by modulating Glu metabolism. RNA sequencing analysis revealed that the mechanism by which targeting TGM2 inhibits drug resistance in Gem-R PDAC cells may be associated with purinergic receptor P2X7 (P2RX7) within the GO:0014049 pathway (positive regulation of glutamate secretion). P2RX7 is highly expressed in Gem-R PDAC cells and tissue samples, and it participates in Glu metabolism and mitophagy in Gem-R PDAC cells. Furthermore, Glu has also been found to induce mitophagy. Lastly, TGM2 and P2RX7 form a positive feedback regulatory loop, jointly regulating Glu metabolism and mitophagy, thereby promoting drug resistance in Gem-R PDAC cells. These data suggest that the TGM2-P2RX7 loop promotes Gem-R in PDAC by improving Glu metabolism and mitophagy, highlighting its potential as a crucial therapeutic target for PDAC.

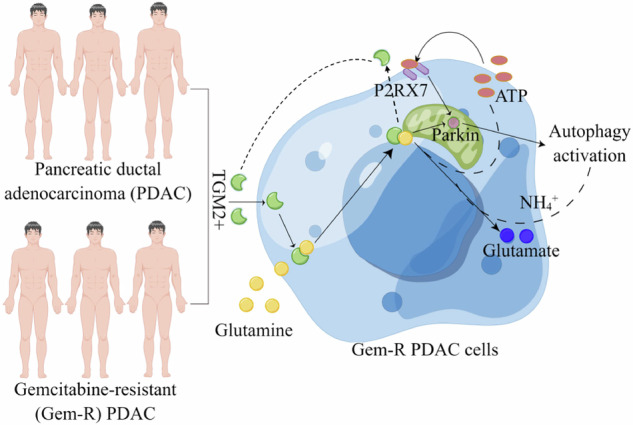

## Introduction

Pancreatic ductal adenocarcinoma (PDAC) is a prevalent and incredibly deadly gastrointestinal cancer, with a survival rate of under 7% [1]. It ranks as the fourth primary cause of cancer-related deaths in the United States [2]. Treatment of PDAC depends on the stage and type of tumor and includes both local (surgery, radiotherapy, and ablative interventions) and systemic (chemotherapy, targeted therapy, and immunotherapy) [3]. While a minority of patients see positive outcomes from existing targeted therapies or immunotherapies, gemcitabine continues to be the primary treatment option for PDAC [4]. Yet, the emergence of drug resistance [5] poses limitations on its effectiveness. Recognizing the significance of gemcitabine in PDAC treatment, it is imperative to delve deeper into the molecular pathways that drive resistance to both PDAC and gemcitabine. This understanding will pave the way for novel therapeutic approaches and enhancements to current treatments for PDAC patients.

Disrupted glutamine (Glu) metabolism stands out as a distinctive feature of cancer cells. The heightened reliance of tumor cells on elevated Glu intake confers them with an unrestricted growth benefit [6, 7]. Transglutaminase type 2 (TGM2), a member of the transglutaminase (TGs) family of Glu transaminases, catalyzes the deamidation or alternatively transamination of specific Gln residues in proteins and peptides [8], and its overexpression is thought to be associated with advanced tumor stage, distant metastasis, and tumor chemoresistance [9]. Functional and clinical validation has shown that higher TGM2 expression is associated with poorer survival, elevated gemcitabine IC50 values, and increased abundance of tumor-infiltrating macrophages in PDAC patients [10]. Therefore, in-depth investigation of TGM2-associated Glu metabolism and gemcitabine resistance (Gem-R) mechanisms may be helpful in the development of neoadjuvant chemotherapy strategies for PDAC.

It is known that extracellular ATP promotes the secretion of TGM2 in macrophages, an effect that can be blocked by selective antagonists of the purinergic receptor P2X7 (P2X7R, also known as P2RX7) [11]. P2X7R, a member of the nucleotide-gated ion channel P2X family, is activated by high concentrations of extracellular ATP [12]. However, TGM2 can, in turn, enhance ATP production by regulating mitochondrial function [13]. Thus, a positive feedback loop involving TGM2-ATP-P2X7R is formed. Furthermore, dysregulation of P2X7R-dependent AMP-activated protein kinase and decreased parkin protein levels can mediate alpha-synuclein-induced mitochondrial mitophagy damage and dysfunction [14]. In mitochondria, Glu is converted to glutamate by glutaminase (GLS), a reaction that produces ammonia. This ammonia enhances autophagy and mitophagy [15]. Intriguingly, upon tumor formation, the activation of mitophagy can induce epithelial-mesenchymal transition, regulate metabolism-particularly Glu utilization and glycolysis-and mediate drug resistance in PDAC [16]. Additionally, P2X7R is found to be highly expressed in PDAC cells, and the P2X7R antagonist AZ10606120 can inhibit cell proliferation [17]. Nevertheless, the interplay and mechanisms of action between TGM2 and P2X7R in Gem-R PDAC require further elucidation.

In this study, we dissected the mechanism of action of targeting TGM2 in Gem-R in PDAC, with the aim of providing a new adjuvant strategy to improve the treatment of PDAC. We verified the correlation between TGM2 expression and Gem-R in PDAC by combining PDAC clinical samples and raw letter analysis. Subsequently, the role of high/low expression of TGM2 in Gem-R PDAC cells was analyzed through gene editing. Finally, RNA sequencing and xenograft tumorigenesis in nude mice were employed to dissect the mechanisms underlying the targeting of TGM2 in Gem-R PDAC. Meanwhile, P2X7R was identified as a key regulator mediating the role of TGM2 in Gem-R in PDAC.

## Results

### High TGM2 expression mediates Gem-R in PDAC

**Fig. 1:**
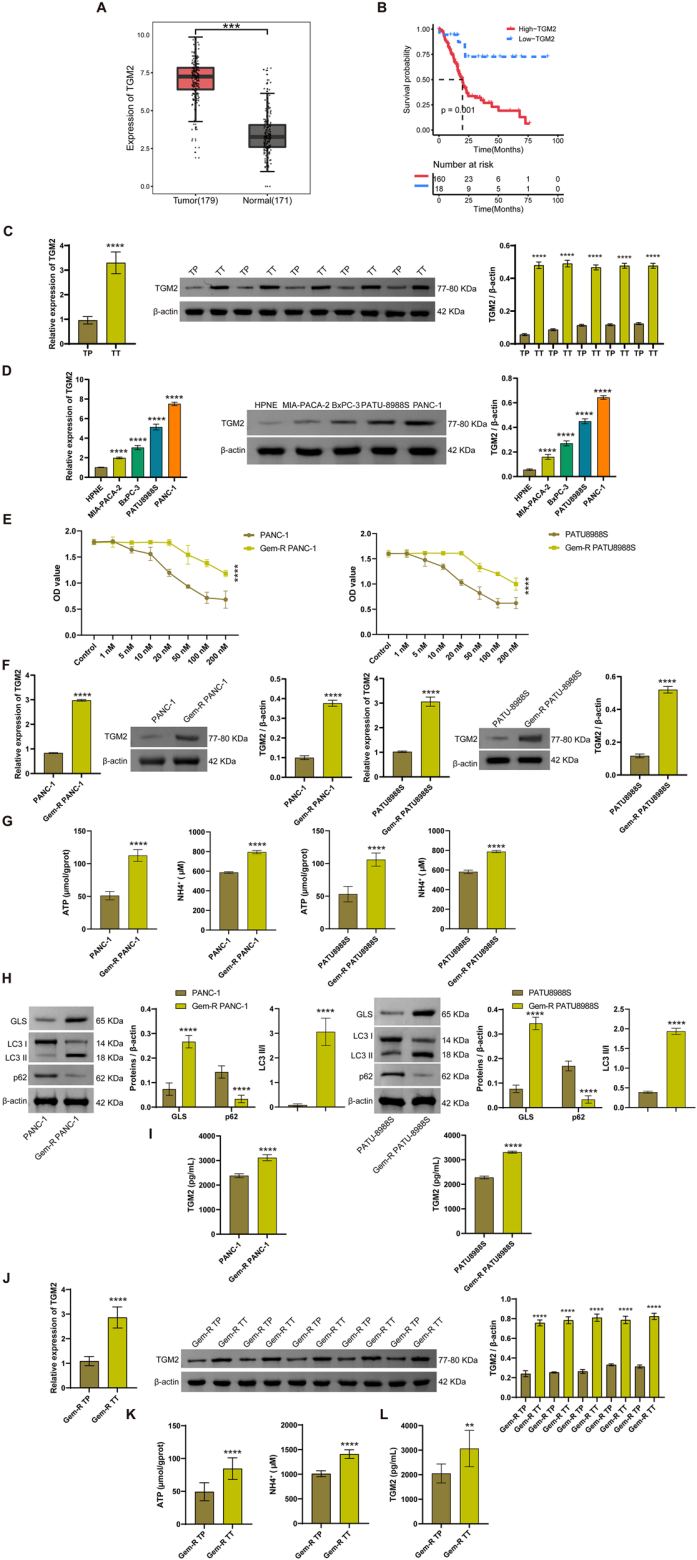
High TGM2 expression mediates Gem-R in PDAC. **A** Box plots show TGM2 expression in 179 PDAC tissues and 171 corresponding non-tumor PDAC tissues. ^***^p < 0.001. **B** Kaplan–Meier curve analysis of the prognostic value of TGM2. **C** RT-qPCR and Western blot analysis of TGM2 expression in TT and TP tissues from PDAC patients. ^****^p < 0.0001 vs. TP. **D** RT-qPCR and Western blot analysis of TGM2 expression in normal pancreatic duct cells (HPNE) and PDAC cells (MIA-PACA-2, BxPC-3, PATU-8988S, PANC-1). ^****^p < 0.0001 vs. HPNE. **E** CCK-8 assay was performed to compare cell viability against PDAC and Gem-R PDAC cells. **F** RT-qPCR and Western blot analysis of TGM2 expression in PDAC and Gem-R PDAC cells. ^****^p < 0.0001 vs. PDAC cells. **G** The levels of ATP and NH_4_^+^ were detected in Gem-R PDAC cells. **H** Western blot analysis of parkin, LC3 Ⅱ/Ⅰ, p62, and GLS expression in Gem-R PDAC cells. **L** The levels of ATP and NH_4_^+^ were detected in Gem-R PDAC cells. **I** The levels of TGM2 in the supernatants of PDAC and Gem-R PDAC cells. **J** RT-qPCR and Western blot analysis of TGM2 expression in TT and TP tissues from PDAC patients with Gem-R. **K** The levels of ATP and NH_4_^+^ were detected in tumor and para-tumor tissues from PDAC patients with Gem-R. **L** The levels of TGM2 were examined in the supernatants of TT and TP tissues from PDAC patients with Gem-R. ^**^p < 0.01, ^****^p < 0.0001 vs. Gem-R TP. *n* = 3.

For the assessment of TGM2 expression in PDAC, analyses were conducted utilizing the TCGA and GTEx databases. The findings indicated a notable upregulation of TGM2 in PDAC samples (Fig. 1A). Furthermore, the correlation between TGM2 expression levels and overall patient survival was investigated by categorizing them into high and low TGM2 scoring groups based on the median score. High TGM2 scores were linked to inferior survival outcomes (Fig. 1B). To corroborate these findings, the expression of TGM2 was examined in TT and TP tissues from PDAC patients. Consistent with the raw letter analysis, TGM2 was highly expressed in TT tissues (Fig. 1C). In addition, TGM2 was highly expressed in PDAC cells (MIA-PACA-2, BxPC-3, PATU-8988S, PANC-1) compared with normal pancreatic ductal cells HPNE (Fig. 1D). To further examine the relationship between TGM2 and Gem-R in PDAC, we constructed Gem-R PDAC cells. We cultured PDAC cells in medium containing increasing concentrations of gemcitabine and maintained them at a concentration of 1 µM gemcitabine, resulting in the generation of chemoresistant cell lines resistant to gemcitabine (Fig. S1). To verify the development of chemoresistance in these cells, we assessed their viability at varying gemcitabine concentrations relative to the parental cells. Our observations revealed that PDAC cells that were continually exposed to gemcitabine exhibited the ability to proliferate at higher gemcitabine concentrations compared to the parental cell lines (Fig. 1E). Furthermore, we investigated the expression levels of TGM2 in Gem-R PDAC cells (PANC-1, PATU-8988S). The results indicated a significant increase in TGM2 expression in Gem-R PDAC cells in comparison to the normal PDAC cells (Fig. 1F). We further analyzed the expression of GLS and levels of Glu-derived metabolites, NH_4_^+^, and ATP. Notably, Gem-R PDAC cells exhibited significantly higher GLS expression and secreted elevated levels of ATP and NH_4_^+^ compared to parental PDAC cells (Fig. 1G, H). Additionally, we examined changes in mitophagy-related proteins. Compared to parental PDAC cells, Gem-R cells showed markedly increased LC3 Ⅱ/Ⅰ ratio and decreased p62 expression (Fig. 1H), indicating enhanced mitophagy activity. Interestingly, Gem-R PDAC cells also secreted significantly higher levels of TGM2 (Fig. 1I). Consistently, tumor tissues from Gem-R PDAC patients demonstrated elevated TGM2 expression (Fig. 1J). Furthermore, Gem-R tumor cells exhibited increased secretion of ATP, NH_4_^+^, and TGM2 compared to adjacent non-tumor tissues (Fig. 1K, L). Collectively, our findings indicate that TGM2 is overexpressed in PDAC and may contribute to Gem-R through modulation of Glu metabolism and mitophagy, accompanied by enhanced TGM2 secretion.

### Silencing or overexpressing TGM2 affects Gem-R, Glu metabolism, and mitophagy in Gem-R PDAC cells

**Fig. 2:**
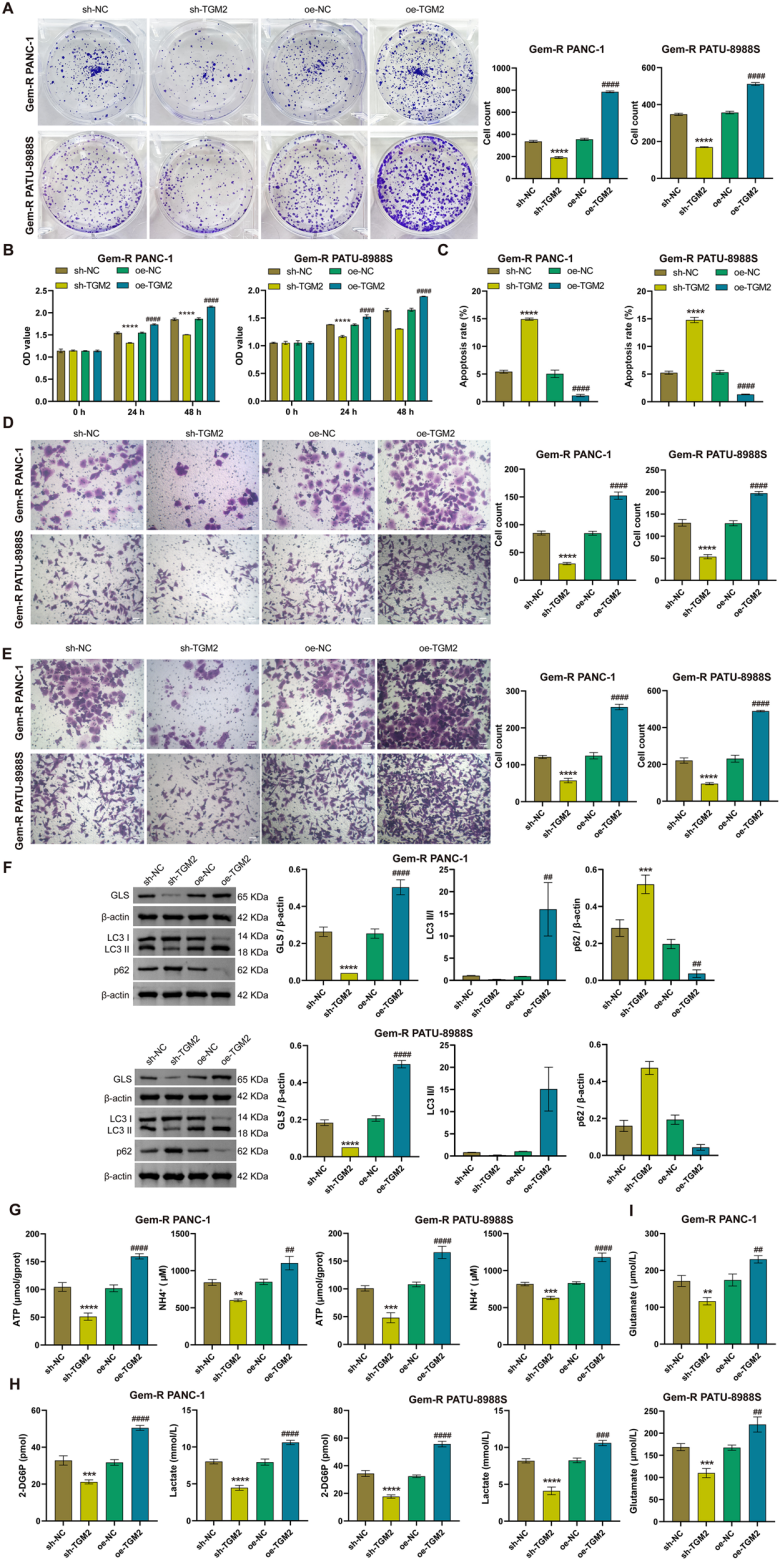
Silencing or overexpressing TGM2 affects Gem-R and Glu metabolism in Gem-R PDAC cells. **A** Clone formation assay for detecting cell proliferation. **B** CCK-8 assay for detecting cell viability. **C** Flow cytometry for detecting cell apoptosis. **D**, **E** Transwell assays for detecting cell invasion and migration. Scale bar: 100 μm. **F** Western blot analysis of GLS, LC3 Ⅱ/Ⅰ, and p62 expression in Gem-R PDAC cells. **G** The levels of ATP and NH_4_^+^ were detected in Gem-R PDAC cells. **H** The levels of 2-DG6P and lactate were detected in Gem-R PDAC cells. **I** The level of glutamate was detected in Gem-R PDAC cells. ^*^p < 0.05, ^**^p < 0.01, ^***^p < 0.001, ^****^p < 0.0001 vs. sh-NC. ^##^*p* < 0.01, ^###^*p* < 0.001, ^####^*p* < 0.0001 vs. oe-NC. *n* = 3.

Our previous results have already confirmed increased ammonia metabolism and ATP secretion in Gem-R PDAC, accompanied by high expression of TGM2. Therefore, we hypothesize that TGM2, as a Glu transferase, is involved in this process. To this end, we constructed TGM2 knockdown and overexpression cell lines by transfecting Gem-R PDAC cells with sh-TGM2 and oe-TGM2, respectively. RT-qPCR and Western blot results confirmed the successful establishment of these cell lines (Fig. S2A, B). Subsequent functional assays revealed that TGM2 knockdown significantly inhibited the proliferation, invasion, and migration of Gem-R PDAC cells, reduced cell viability, and promoted apoptosis (Figs. 2A–E and S2C). Conversely, overexpression of TGM2 exhibited completely opposite trends (Fig. 2A–E). To verify the impact of TGM2 on Glu metabolism in Gem-R PDAC cells, we examined changes in related indicators. The results showed that TGM2 knockdown decreased the expression of mitochondrial GLS and reduced the levels of ATP, NH_4_^+^, glucose, lactate, and glutamate (Fig. 2F–I). However, overexpression of TGM2 increased GLS expression and elevated the levels of ATP, NH_4_^+^, glucose, lactate, and glutamate (Fig. 2F–I). In addition, the expression levels of the mitophagy-related proteins LC3 II/Ⅰ were decreased, and the expression levels of p62 were increased in the sh-TGM2 group compared with the NC group (Fig. 2F). In summary, these findings indicate that silencing TGM2 inhibits Gem-R, Glu metabolism, and mitophagy in Gem-R PDAC cells, while overexpressing TGM2 promotes these processes.

### Silencing TGM2 blocks Glu-induced Gem-R, glutamate secretion, and mitophagy in Gem-R PDAC

**Fig. 3:**
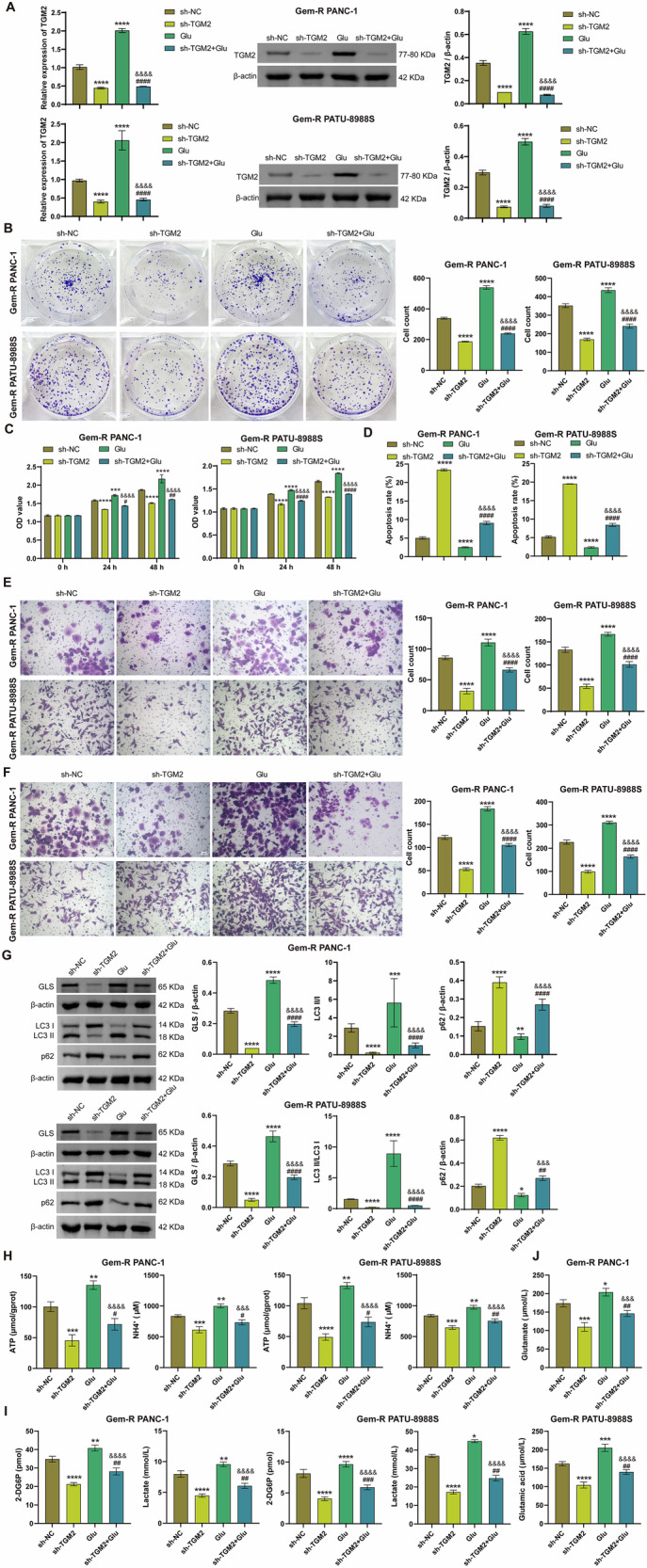
Silencing TGM2 blocks Glu-induced Gem-R and Glu metabolism in Gem-R PDAC cells. **A** RT-qPCR and Western blot analysis of TGM2 expression in Gem-R PDAC cells. **B** Clone formation assay to detect cell proliferation. Scale bar: 100 μm. **C**. CCK-8 assay to detect cell viability. **D** Flow cytometry to detect cell apoptosis. **E**, **F** Transwell assay to detect cell invasion and migration. Scale bar: 100 μm. **G** Western blot analysis of GLS, LC3 Ⅱ/Ⅰ, and p62 expression in Gem-R PDAC cells. **H** The levels of ATP and NH_4_^+^ were detected in Gem-R PDAC cells. **I** The levels of 2-DG6P and lactate were detected in Gem-R PDAC cells. **J** The level of glutamate was detected in Gem-R PDAC cells. ^*^p < 0.05, ^**^p < 0.01, ^***^p < 0.001, ^****^p < 0.0001 vs. sh-NC. ^#^*p* < 0.05, ^##^*p* < 0.01, ^###^p < 0.001, ^####^*p* < 0.0001 vs. sh-TGM2. ^&&&^*p* < 0.001, ^&&&&^*p* < 0.0001 vs. Glu. *n* = 3.

**Fig. 4:**
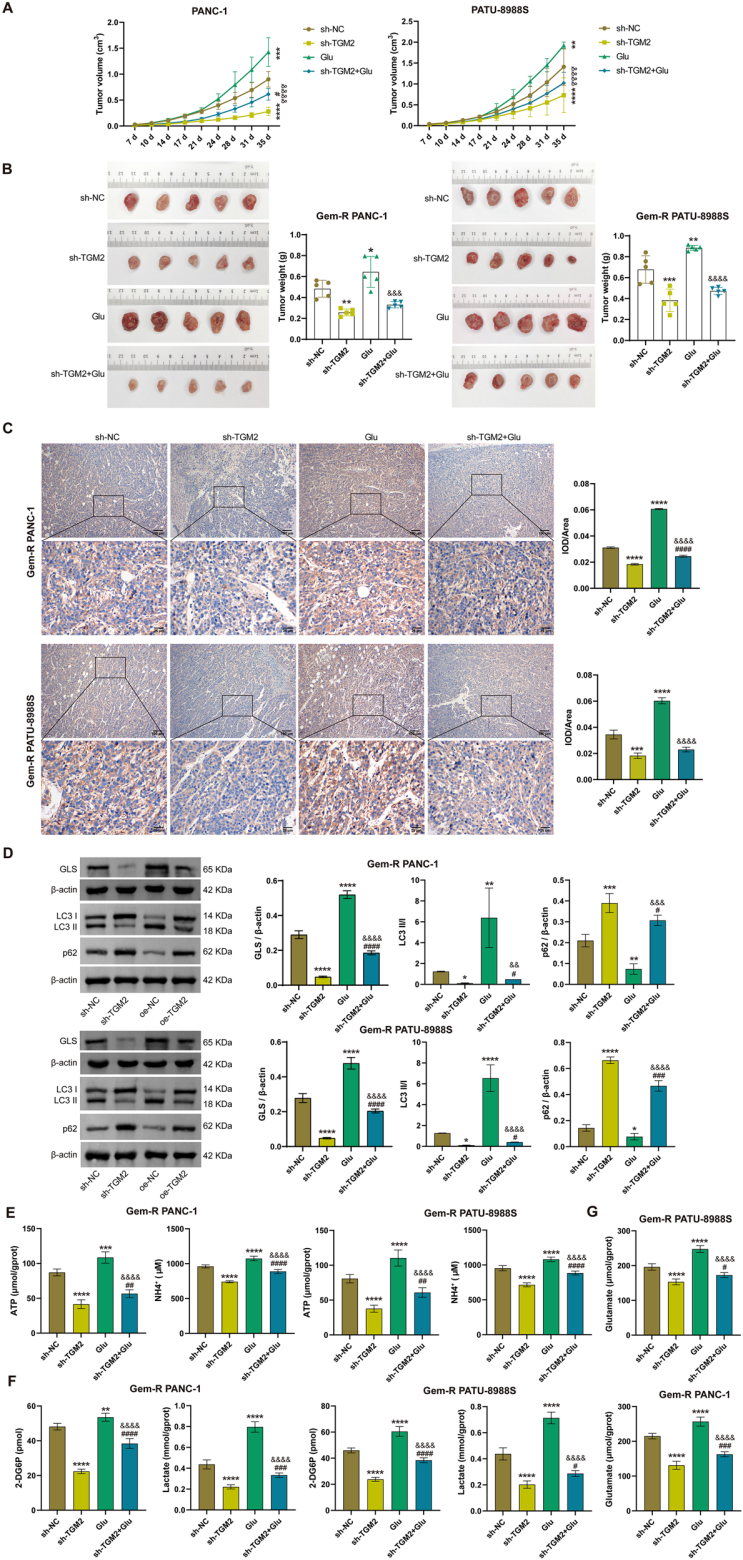
Silencing TGM2 blocks Glu-induced drug resistance and glutamate secretion in Gem-R PDAC mice. **A** Growth curves of tumors in nude mice. **B** Images of tumors and diagrams of tumor weight. **C** IHC assay to detect TGM2 expression in tumor tissues. Scale bar: 100 μm and 25 μm. **D** Western blot analysis of GLS, LC3 Ⅱ/Ⅰ, and p62 expression in tumor tissues. **E** The levels of ATP and NH_4_^+^ were detected in tumor tissues. **F** The levels of 2-DG6P and lactate were detected in tumor tissues. **G** The level of glutamate was detected in tumor tissues. ^*^p < 0.05, ^**^p < 0.01, ^***^p < 0.001, ^****^p < 0.0001 vs. sh-NC. ^#^*p* < 0.05, ^##^*p* < 0.01, ^###^*p* < 0.001, ^####^*p* < 0.0001 vs. sh-TGM2. ^&&&^*p* < 0.001, ^&&&&^*p* < 0.0001 vs. Glu. *n* = 5.

To investigate whether TGM2 affects the sensitivity of Gem-R PDAC cells to gemcitabine and the levels of mitophagy by regulating Glu metabolism, we constructed a TGM2 knockdown cell line and pretreated the cells with Glu. RT-qPCR and Western blot results showed that sh-TGM2 decreased the expression of TGM2 and Glu increased its expression (Fig. 3A). As shown in Figs. 3B–F and S2D, Glu treatment significantly promoted the proliferation, invasion, and migration of Gem-R PDAC cells, enhanced cell viability, and inhibited apoptosis. Notably, sh-TGM2 partially abrogated these effects of Glu. Furthermore, Glu also markedly upregulated the expression of GLS and LC3 Ⅱ/Ⅰ, downregulated the expression of p62, and elevated the levels of ATP, NH_4_^+^, glucose, lactate, and glutamate, and these effects were alleviated by sh-TGM2 (Fig. 3G–J). These results suggest that silencing TGM2 blocks Glu-induced drug resistance, Glu metabolism, and mitophagy in Gem-R PDAC cells. In addition, we constructed a Gem-R PDAC mouse model by subcutaneously injecting Gem-R PDAC cells transfected with sh-NC or sh-TGM2 into nude mice with Glu. Obviously, compared to the sh-NC group, tumor growth was significantly inhibited in the sh-TGM2 group, while tumor growth was more rapid in mice treated with Glu, and sh-TGM2 suppressed the tumor-promoting effect of Glu (Fig. 4A, B). Meanwhile, Immunohistochemistry (IHC) results from mouse tumor tissues showed that the expression of TGM2 was significantly reduced in the sh-TGM2 group compared to the sh-NC group, and increased markedly in the Glu group (Fig. 4C). Compared to the Glu group, the expression of TGM2 was decreased in the sh-TGM2+Glu group (Fig. 4C). Furthermore, consistent with the in vitro experiments, sh-TGM2 significantly downregulated the expression of GLS and LC3 Ⅱ/Ⅰ, upregulated the expression of p62, and decreased the levels of ATP, NH_4_^+^, glucose, lactate, and glutamate. In contrast, Glu exhibited opposite effects, and these effects were partially abolished by sh-TGM2 (Fig. 4D–G). These results indicate that silencing TGM2 blocks Glu-induced tumor growth, glutamate secretion, and mitophagy.

### RNA sequencing indicates that the GO:0014049 pathway is associated with the regulation of glutamate secretion by TGM2 in Gem-R PDAC cells

**Fig. 5:**
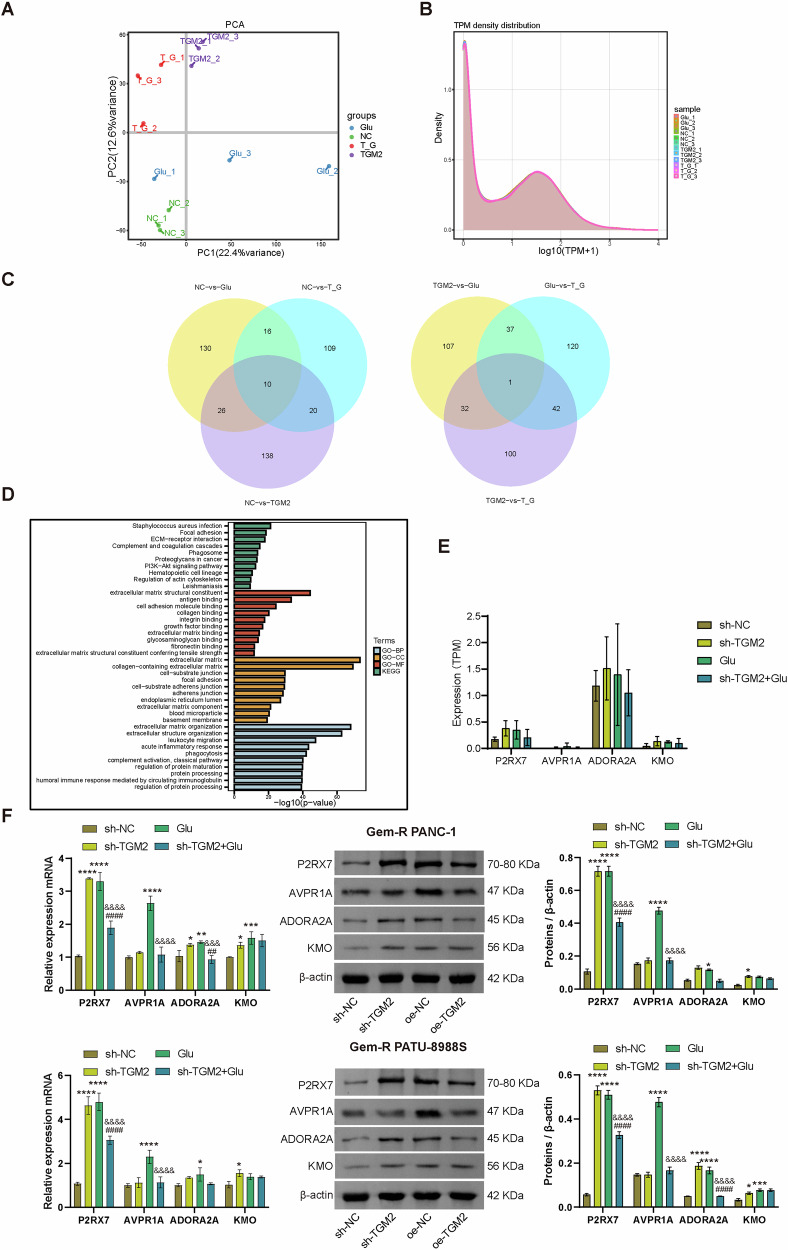
RNA sequencing indicates that the GO:0014049 pathway is associated with the regulation of glutamate secretion by TGM2 in Gem-R PDAC cells. **A** PCA analysis showed differences between groups. **B** TPM curves showed sequencing stability. **C** Venn diagram showing intersection of mitophagy pathway-related DEGs in different combinations of intergroup comparisons (sh-NC vs. Sh-TGM2, sh-TGM2 vs. sh-TGM2+Glu, Glu vs. sh-TGM2, sh-NC vs. Glu). **D** KEGG and GO analysis. **E** The expression analysis of P2RX7, AVPR1A, ADORA2A, and KMO by RNA sequencing. **F** RT-qPCR and Western blot analysis of P2RX7, AVPR1A, ADORA2A, and KMO expression in Gem-R PDAC cells. ^*^p < 0.05, ^**^p < 0.01, ^***^p < 0.001, ^****^p < 0.0001 vs. sh-NC. ^##^*p* < 0.01, ^####^*p* < 0.0001 vs. sh-TGM2. ^&&&^*p* < 0.001, ^&&&&^*p* < 0.0001 vs. Glu. *n* = 3.

To further elucidate the mechanism by which TGM2 regulates Glu metabolism in Gem-R PDAC cells, we employed RNA sequencing combined with KEGG and GO databases to analyze Glu-related pathways. Principal component analysis (PCA) was conducted based on differentially expressed genes (DEGs) between groups. As shown in the figure, there are clear differences among the different treatment groups (Fig. 5A). The Transcripts Per Million (TPM) plot illustrates the distribution of gene expression levels across different samples, demonstrating the stability of the sequencing (Fig. 5B). Additionally, a Venn diagram displays the intersections of DEGs among different groups. Among the DEGs comparing the sh-NC group with the sh-TGM2, Glu, and sh-TGM2+Glu groups, there are 10 common DEGs, including CMTM1, SH3BP1, ZC4H2, CTD-2369P2.10, HIST1H3J, CFB, TRIM39-RPP21, MRPS17, NT5C1B-RDH14, and TM9SF1 (Fig. 5C, left). In contrast, only one common DEG, TEN1, is found among the DEGs comparing sh-TGM2 vs. Glu, sh-TGM2 vs. sh-TGM2+Glu, and Glu vs. sh-TGM2+Glu (Fig. 5C, right). KEGG and GO analyses revealed that while there were no significant changes in KEGG enrichment, notable variations were observed in GO enrichment, with the positive regulation of glutamate secretion (GO:0014049) pathway exhibiting significant changes among groups (Fig. 5D). In the comparison between sh-NC and sh-TGM2, the DEGs enriched in the GO:0014049 pathway included P2RX7, AVPR1A, ADORA2A, and KMO (Table 1). To further investigate the key gene mediating the role of TGM2, we analyzed the expression of these genes across different groups based on RNA sequencing results and validated them through experiments (Fig. 5E, F). The results showed that P2RX7 exhibited the most significant intergroup expression difference. Compared to the sh-NC group, the expression of P2RX7 was upregulated in both the sh-TGM2 and Glu groups. However, when compared to the sh-TGM2 and Glu groups, P2RX7 was downregulated in the sh-TGM2+Glu group. These findings suggest that P2RX7 may be involved in the regulation of glutamate secretion by TGM2 in Gem-R PDAC cells, and its expression is influenced by both TGM2 and Glu.

**Table 1:** GO:0014049 pathway enriched for DEGs.

ID	Description	Set Size	enrichmentScore	*P* value	core_enrichment
GO:0014049	Positive regulation of glutamate secretion	12	0.846	0.0008	NC-vs-Glu: KMO/SYT4/P2RX7/AVPR1A/ADORA2A/AVP/AVPR1B
		12	0.765	0.0168	NC-vs-TGM2: P2RX7/AVP/AVPR1A/ADORA2A/KMO
		12	−0.735	0.0345	Glu-vs-TGM2: KMO/SYT4/AVPR1A/AVPR1B/P2RX7/ADORA2A

### P2RX7 is involved in Glu metabolism and mitophagy in Gem-R PDAC cells

**Fig. 6:**
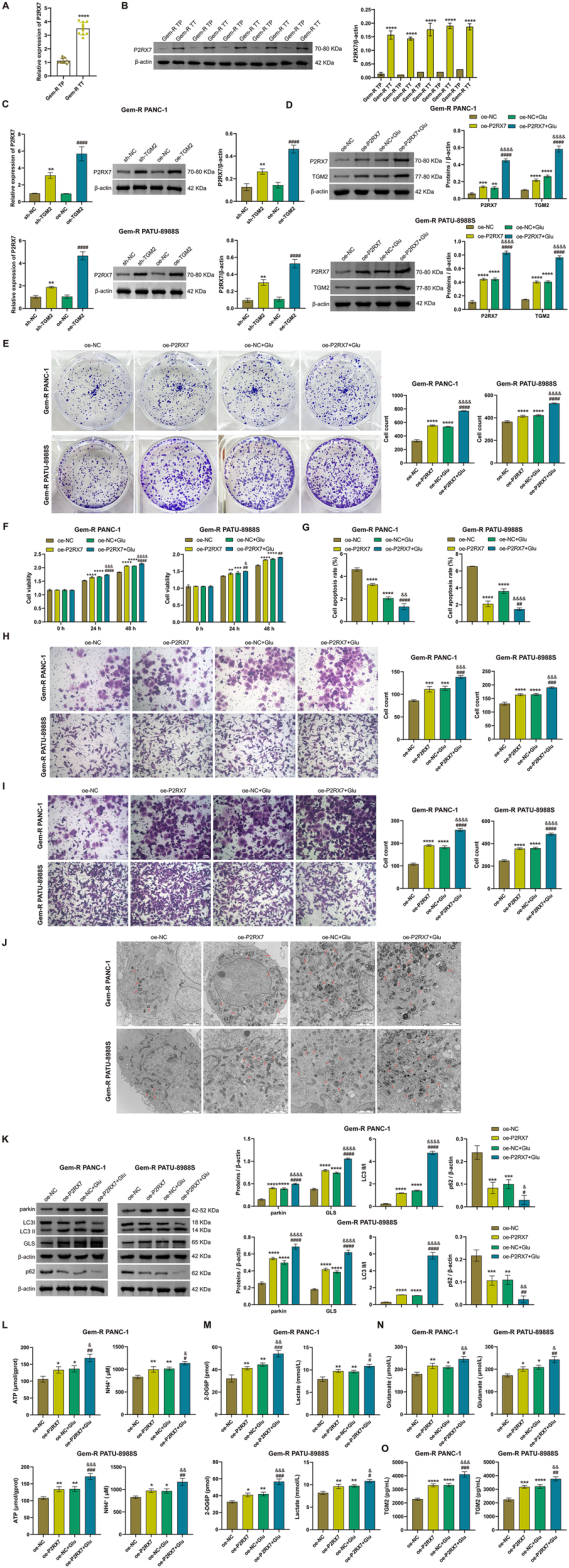
P2RX7 is involved in Glu metabolism and mitophagy in Gem-R PDAC cells. **A**, **B** RT-qPCR and Western blot analysis of P2RX7 expression in PDAC and para-tumor tissues from PDAC patients with Gem-R. ^****^p < 0.0001 vs. Gem-R TP. **C** Western blot analysis of P2RX7 expression in Gem-R PDAC cells. ^**^p < 0.01 vs. sh-NC, ^####^*p* < 0.0001 vs. oe-NC. **D** Western blot analysis of P2RX7 and TGM2 expression in Gem-R PDAC cells. **E** Clone formation assay to detect cell proliferation. Scale bar: 100 μm. **F** CCK-8 assay to detect cell viability. **G** Flow cytometry to detect cell apoptosis. **H**, **I** Transwell assay to detect cell invasion and migration. Scale bar: 100 μm. **J** Representative TEM images of an autophagosome (arrow) in Gem-R PDAC cells. Scale bar: 2 μm. **K** Western blot analysis of parkin, LC3 Ⅱ/Ⅰ, p62, and GLS expression in Gem-R PDAC cells. **L** The levels of ATP and NH_4_^+^ were detected in Gem-R PDAC cells. **M** The levels of 2-DG6P and lactate were detected in Gem-R PDAC cells. **N** The level of glutamate was detected in Gem-R PDAC cells. **O** The levels of TGM2 in the supernatants of Gem-R PDAC cells. ^*^p < 0.05, ^**^p < 0.01, ^***^p < 0.001, ^****^p < 0.0001 vs. oe-NC. ^#^*p* < 0.05, ^##^*p* < 0.01, ^###^*p* < 0.001, ^####^*p* < 0.0001 vs. oe-P2RX7. ^&^*p* < 0.05, ^&&^*p* < 0.01, ^&&&^*p* < 0.001, ^&&&&^*p* < 0.0001 vs. Glu. *n* = 3.

To further investigate the role of P2RX7 in Glu metabolism, we conducted additional studies. Compared to adjacent non-tumor tissues, tumor tissues from Gem-R PDAC patients exhibited high expression of P2RX7, suggesting that P2RX7 may serve as an oncogenic factor in Gem-R PDAC (Fig. 6A, B). Additionally, compared with the NC group, P2RX7 expression was upregulated in both the sh-TGM2 group and, notably, the oe-TGM2 group (Fig. 6C). This suggests that TGM2 can regulate P2RX7 expression. Subsequently, we established a Gem-R PDAC cell line overexpressing P2RX7 (Fig. S2E). In Gem-R PDAC cells, both oe-P2RX7 and Glu treatment increased the expression of P2RX7 and TGM2 (Fig. 6D). Cellular functional experiments demonstrated that both oe-P2RX7 and Glu promoted cell proliferation, invasion, and migration, enhanced cell viability, and inhibited apoptosis in Gem-R PDAC cells (Figs. 6E–I and S2F). Furthermore, numerous studies have highlighted the pivotal role of P2X7R in mitophagy regulation [18, 19]. Therefore, we also examined mitophagy-related indicators. Transmission electron microscopy (TEM) analysis revealed that overexpression of P2X7R and Glu increased the formation of autophagosomes in Gem-R PDAC cells (Fig. 6J). Western blot results demonstrated that overexpression of P2X7 receptor and glutamate increased the levels of mitophagy markers Parkin and the LC3 II/ I ratio, while decreasing p62 levels (Fig. 6K). These findings indicate that both P2X7R and Glu promote mitophagy in Gem-R PDAC cells. Additionally, overexpression of P2X7R increased the expression of GLS, enhanced the levels of ATP, NH_4_^+^, glucose, lactate, glutamate, and extracellular TGM2, and further potentiated the effects of Glu (Fig. 6K–O). In summary, P2RX7 is involved in Glu metabolism and mitophagy in Gem-R PDAC cells and promotes TGM2 expression and secretion.

### ATP promotes TGM2 secretion via P2RX7, enhancing Glu metabolism and mitophagy in Gem-R PDAC cells

**Fig. 7:**
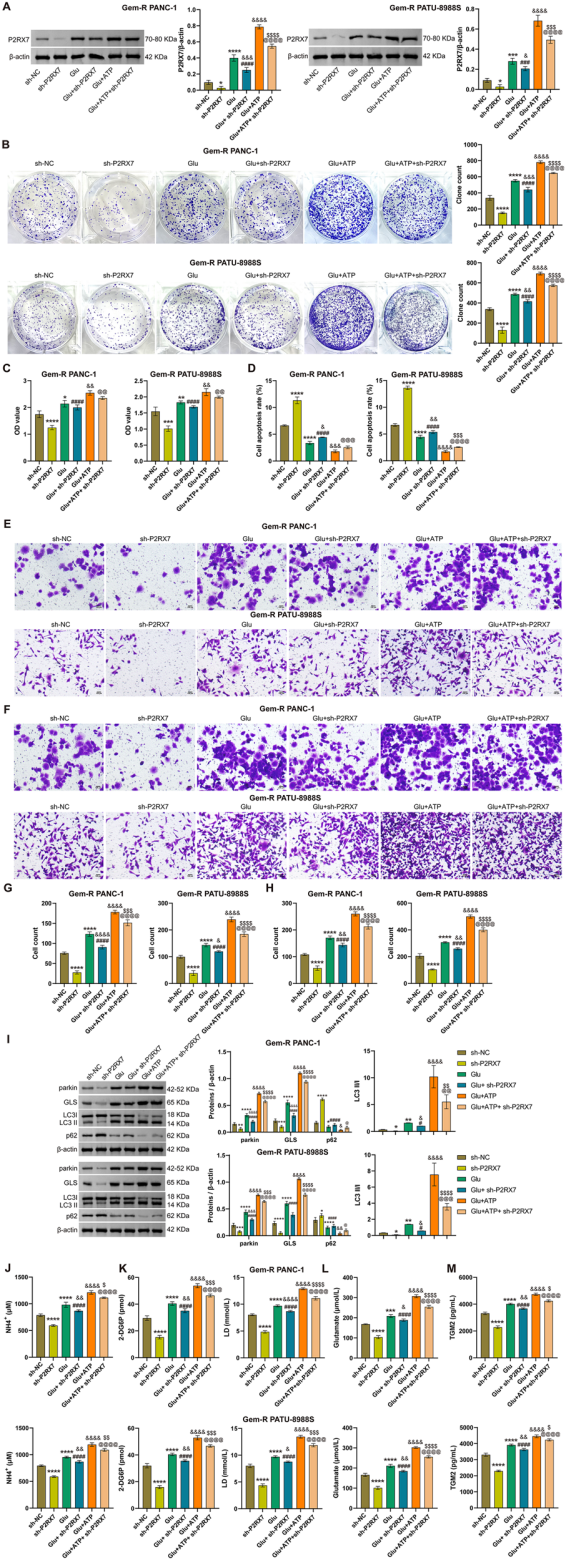
ATP promotes TGM2 secretion via P2RX7, inducing Glu metabolism and mitophagy in Gem-R PDAC cells. **A**. Western blot analysis of P2RX7 expression in Gem-R PDAC cells. **B** Clone formation assay to detect cell proliferation. Scale bar: 100 μm. **C** CCK-8 assay to detect cell viability. **D** Flow cytometry to detect cell apoptosis. **E**, **G** Transwell assay to detect cell invasion. Scale bar: 100 μm. **F**, **H** Transwell assay to detect cell migration. Scale bar: 100 μm. **I** Western blot analysis of parkin, LC3 Ⅱ/Ⅰ, p62, and GLS expression in Gem-R PDAC cells. **J** The levels of ATP and NH_4_^+^ were detected in Gem-R PDAC cells. **K** The levels of 2-DG6P and lactate were detected in Gem-R PDAC cells. **L** The level of glutamate was detected in Gem-R PDAC cells. **M** The levels of TGM2 in the supernatants of Gem-R PDAC cells. ^*^p < 0.05, ^**^p < 0.01, ^***^p < 0.001, ^****^p < 0.0001 vs. sh-NC. ^#^*p* < 0.05, ^###^*p* < 0.001, ^####^*p* < 0.0001 vs. sh- P2RX7. ^&^p < 0.05, ^&&^*p* < 0.01, ^&&&^*p* < 0.001, ^&&&&^*p* < 0.0001 vs. Glu. ^@^*p* < 0.05, ^@@^*p* < 0.01, ^@@@^*p* < 0.001, ^@@@@^*p* < 0.0001 vs. Glu+sh-P2RX7. ^$^*p* < 0.05, ^$$^*p* < 0.01, ^$$$^p < 0.001, ^$$$$^*p* < 0.0001 vs. Glu+ATP. *n* = 3.

Building on our previous findings that P2RX7 is involved in Glu metabolism, mitophagy, and TGM2 secretion in Gem-R PDAC cells, and given that extracellular ATP has been reported to stimulate macrophage TGM2 secretion through P2X7R activation (a process inhibitable by selective P2X7R antagonists) [11], we hypothesized that ATP might promote TGM2 secretion via P2RX7 to sustain Gem-R-associated Glu metabolism in PDAC. To test this hypothesis, we first established P2RX7-knockdown Gem-R PDAC cell lines using shRNA, selecting the most efficient sh-P2RX7 construct for subsequent experiments (Fig. S2G). Gem-R PDAC cells with or without P2RX7 knockdown were treated with Glu and/or ATP. Western blot analysis revealed that sh-P2RX7 effectively reduced P2RX7 expression, while both Glu and ATP treatments significantly upregulated P2RX7 levels (Fig. 7A). Functional assays demonstrated that P2RX7 knockdown markedly suppressed Gem-R PDAC cell proliferation, invasion, migration, and viability, while promoting apoptosis (Figs. 7B–H and S3A). Notably, ATP enhanced the Glu-induced promotion of these malignant phenotypes and counteracted the inhibitory effects of P2RX7 knockdown. Mechanistic investigations showed that P2RX7 knockdown downregulated mitophagy markers (Parkin, LC3Ⅱ/Ⅰ ratio) and upregulated p62 levels compared to controls. Glu/ATP co-treatment further increased Parkin and LC3Ⅱ/Ⅰ expression while decreasing p62 levels, effects that were abrogated by P2RX7 knockdown (Fig. 7I). Metabolic analysis revealed that P2RX7 knockdown reduced GLS expression and decreased extracellular levels of ATP, NH_4_^+^, glucose, lactate, glutamate, and TGM2 (Fig. 7I–M). Glu/ATP co-treatment synergistically enhanced GLS expression and the secretion of these metabolites/proteins, reversing the inhibitory effects of P2RX7 knockdown. Collectively, these data demonstrate that ATP promotes Gem-R in PDAC cells by upregulating P2RX7 expression, thereby activating a metabolic-secretory axis characterized by enhanced Glu metabolism, mitophagy, and TGM2 secretion.

### TGM2 activates P2RX7 through enhancement of Glu metabolism and ATP secretion, thereby promoting mitophagy and Gem-R

**Fig. 8:**
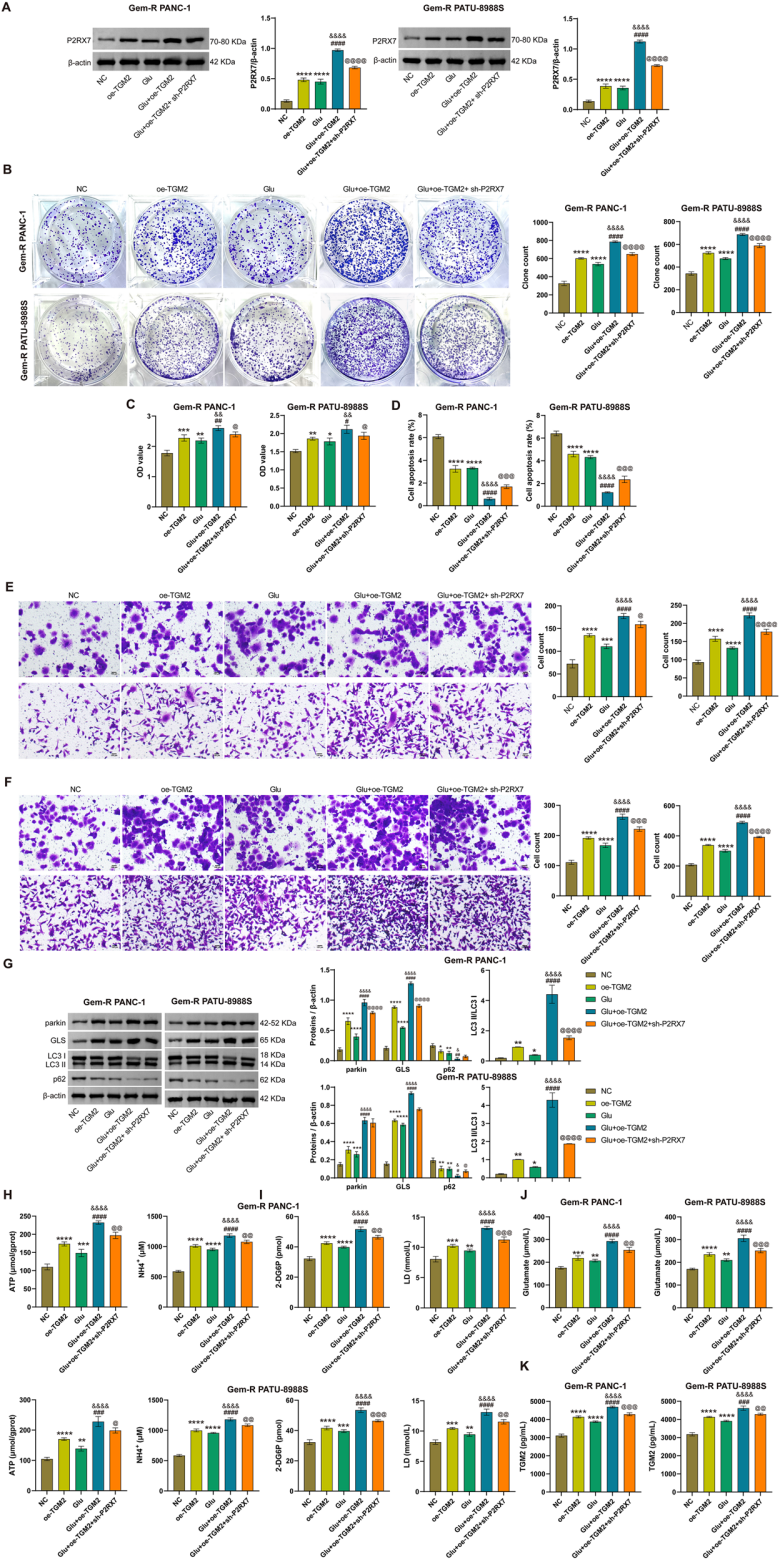
TGM2 activates P2RX7 through enhancement of Glu metabolism and ATP secretion, thereby promoting mitophagy and Gem-R. **A** Western blot analysis of P2RX7 expression in Gem-R PDAC cells. **B** Clone formation assay to detect cell proliferation. Scale bar: 100 μm. **C** CCK-8 assay to detect cell viability. **D** Flow cytometry to detect cell apoptosis. **E**, **F** Transwell assay to detect cell invasion and migration. Scale bar: 100 μm. **G** Western blot analysis of parkin, LC3 Ⅱ/Ⅰ, p62, and GLS expression in Gem-R PDAC cells. **H** The levels of ATP and NH_4_^+^ were detected in Gem-R PDAC cells. **I** The levels of 2-DG6P and lactate were detected in Gem-R PDAC cells. **J** The level of glutamate was detected in Gem-R PDAC cells. **K** The levels of TGM2 in the supernatants of Gem-R PDAC cells. ^*^p < 0.05, ^**^p < 0.01, ^***^p < 0.001, ^****^p < 0.0001 vs. NC. ^#^p < 0.05, ^##^*p* < 0.01, ^###^*p* < 0.001, ^####^*p* < 0.0001 vs. oe-TGM2. ^&^*p* < 0.05, ^&&^*p* < 0.01, ^&&&^*p* < 0.001, ^&^^&&&^*p* < 0.0001 vs. Glu. ^@^*p* < 0.05, ^@@^*p* < 0.01, ^@@@^*p* < 0.001, ^@@@@^*p* < 0.0001 vs. Glu+oe-TGM2. *n* = 3.

Our findings revealed a bidirectional regulatory axis between TGM2 and P2RX7 in Gem-R PDAC. We performed gain- and loss-of-function studies. Overexpression of TGM2 (oe-TGM2) and Glu treatment significantly upregulated P2RX7 expression, an effect partially reversed by P2RX7 knockdown (sh-P2RX7) (Fig. 8A). To further investigate the regulatory mechanism between TGM2 and P2RX7, we employed dual-luciferase reporter assays and Co-IP experiments for evaluation. The results demonstrated that neither overexpression nor knockdown of TGM2 significantly altered P2RX7 promoter activity, and no direct interaction between TGM2 and P2RX7 proteins was detected (Fig. S4A, B). These findings indicate that TGM2 does not regulate P2RX7 transcription nor directly act upon the P2RX7 protein. Building on the established role of TGM2 in promoting Glu metabolism and ATP production, we hypothesized that TGM2-mediated metabolic reprogramming activates P2RX7 through extracellular ATP secretion, creating a self-reinforcing loop that sustains chemoresistance. Functional assays demonstrated that oe-TGM2 and Glu synergistically enhanced Gem-R PDAC cell proliferation, invasion, migration, and viability while suppressing apoptosis. These pro-malignant effects were significantly attenuated by sh-P2RX7 (Figs. 8B–F and S3B). Western blot analysis confirmed these observations: oe-TGM2 and Glu treatment elevated mitophagy markers (Parkin, LC3Ⅱ/Ⅰ ratio) and reduced p62 levels. The combination of oe-TGM2 and Glu further amplified these changes, which were partially reversed by sh-P2RX7 (Fig. 8G). Metabolic profiling showed that oe-TGM2 and Glu treatment increased GLS expression and secretion of ATP, NH_4_^+^, glucose, lactate, glutamate, and extracellular TGM2. P2RX7 knockdown significantly attenuated these metabolic alterations (Fig. 8H–K). Additionally, we employed CBX, a selective inhibitor of Pannexin1 (Panx1, an ATP-release channel), to investigate its effects. Notably, CBX treatment significantly reduced ATP release from Gem-R PDAC cells and abolished TGM2-mediated regulation of ATP secretion, as evidenced by experimental data (Fig. S4C). Concurrently, CBX downregulated P2RX7 expression and attenuated TGM2’s regulatory influence on P2RX7 activity (Fig. S4D). Taken together, TGM2 promotes mitochondrial autophagy and Gem-R by promoting Glu metabolism and ATP secretion to activate P2RX7.

### TGM2-P2RX7 loop regulates Glu metabolism and mitophagy, promoting Gem-R in Gem-R PDAC cells

**Fig. 9:**
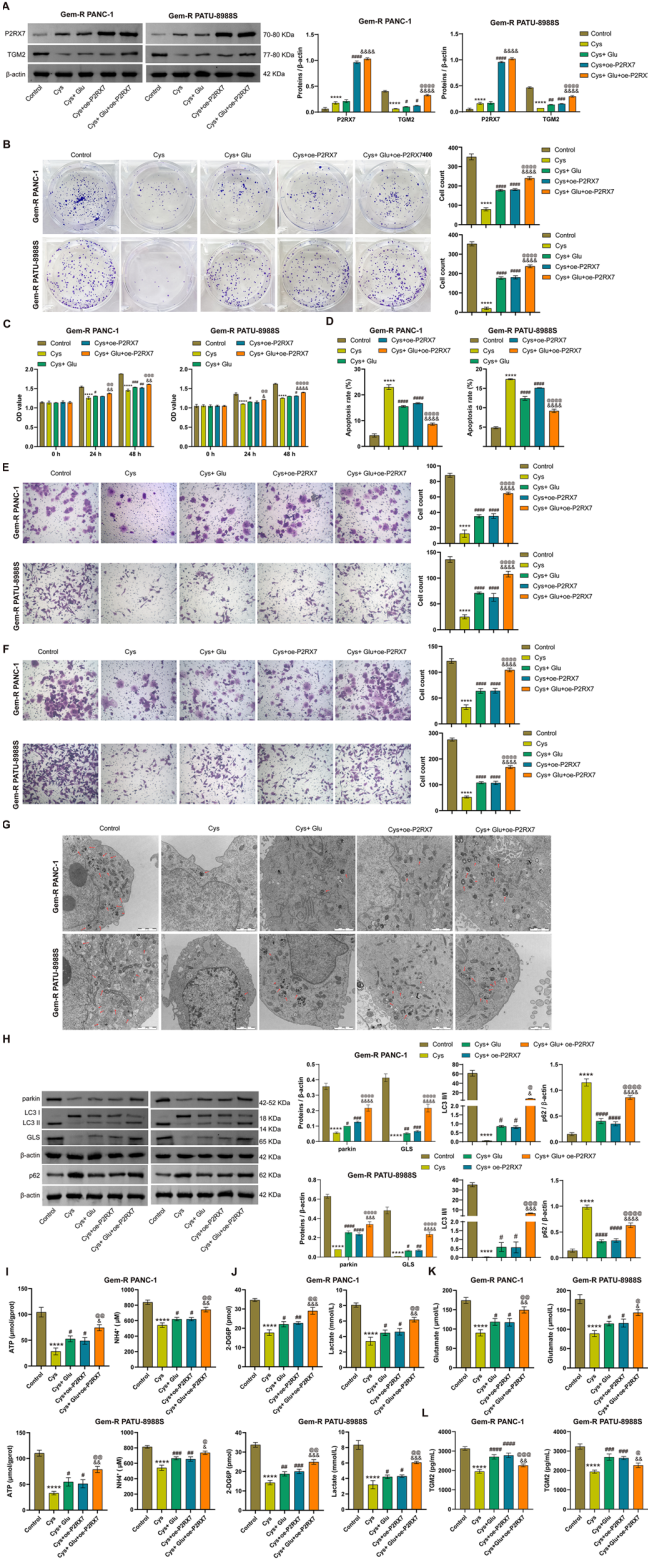
TGM2-P2RX7 loop regulates Glu metabolism and mitophagy, promoting Gem-R in Gem-R PDAC cells. **A** Western blot analysis of TGM2 and P2RX7 expression in Gem-R PDAC cells. **B** Clone formation assay to detect cell proliferation. Scale bar: 100 μm. **C** CCK-8 assay to detect cell viability. **D** Flow cytometry to detect cell apoptosis. **E**, **F**. Transwell assay to detect cell invasion and migration. Scale bar: 100 μm. **G** Representative TEM images of an autophagosome (arrow) in Gem-R PDAC cells. Scale bar: 2 μm. **H** Western blot analysis of parkin, LC3 Ⅱ/Ⅰ, p62, and GLS expression in Gem-R PDAC cells. **I** The levels of ATP and NH_4_^+^ were detected in Gem-R PDAC cells. **J** The levels of 2-DG6P and lactate were detected in Gem-R PDAC cells. **K** The level of glutamate was detected in Gem-R PDAC cells. **L** The levels of TGM2 in the supernatants of Gem-R PDAC cells. ^****^p < 0.0001 vs. Control. ^#^*p* < 0.05, ^##^*p* < 0.01, ^###^*p* < 0.001, ^####^*p* < 0.0001 vs. Cys. ^&^*p* < 0.05, ^&&^*p* < 0.01, ^&&&^*p* < 0.001, ^&&&&^*p* < 0.0001 vs. Cys+Glu. ^@^*p* < 0.05, ^@@^*p* < 0.01, ^@@@^*p* < 0.001, ^@@@@^*p* < 0.0001 vs. Cys+oe-P2RX7. *n* = 3.

**Fig. 10:**
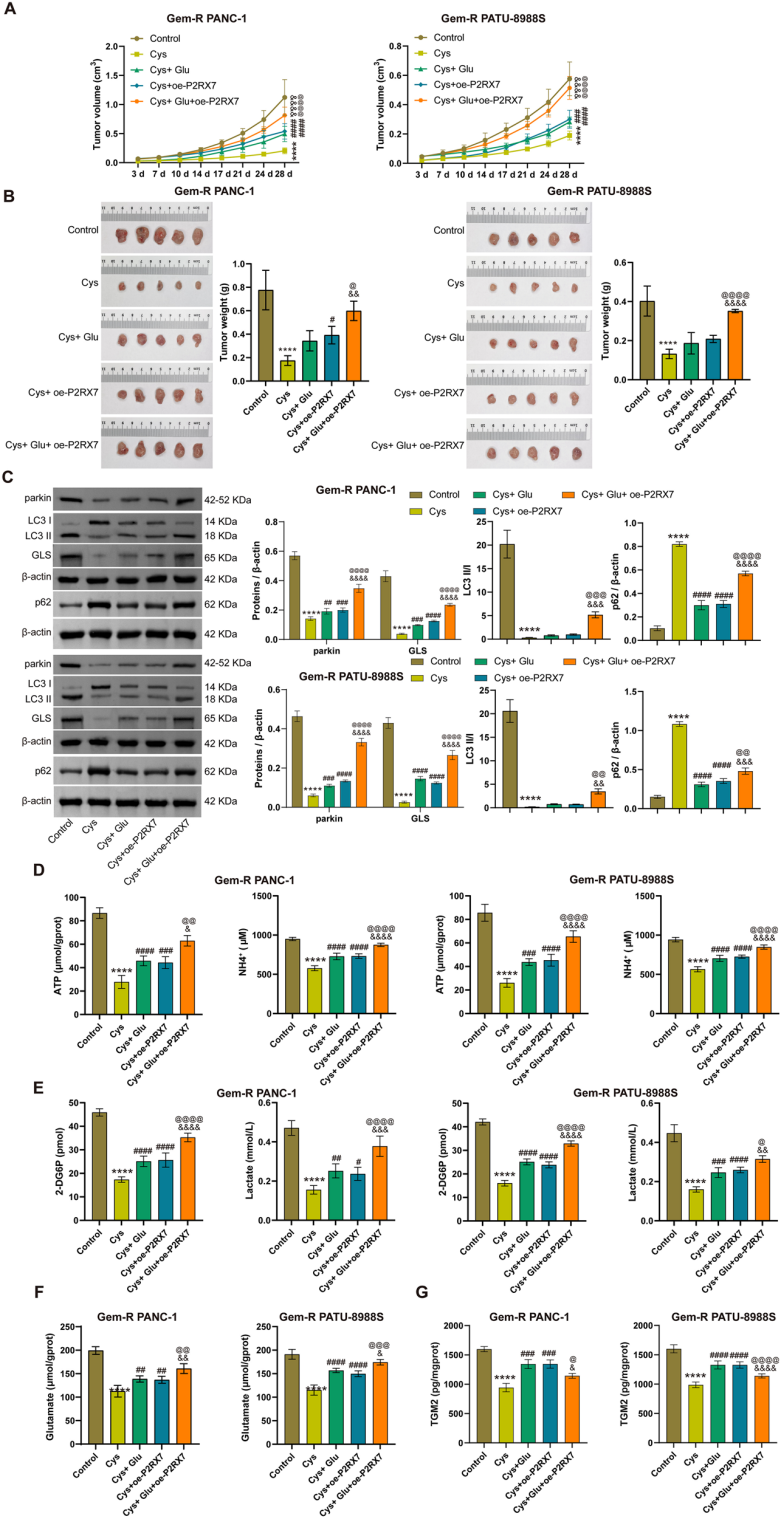
TGM2-P2RX7 loop regulates Glu metabolism and mitophagy, promoting drug resistance in Gem-R PDAC mice. **A** Growth curves of tumors in nude mice. **B** Images of tumors and diagrams of tumor weight. **C** Western blot analysis of parkin, LC3 Ⅱ/Ⅰ, p62, and GLS expression in tumor tissues. **D** The levels of ATP and NH_4_^+^ were detected in tumor tissues. **E** The levels of 2-DG6P and lactate were detected in tumor tissues. **F** The level of glutamate was detected in tumor tissues. **G** The levels of TGM2 were examined in the supernatants of tumor tissues. ****p < 0.0001 vs. Control. ^#^*p* < 0.05, ^##^*p* < 0.01, ^###^*p* < 0.001, ####*p* < 0.0001 vs. Cys. ^&^*p* < 0.05, ^&&^*p* < 0.01, ^&&&^*p* < 0.001, ^&&&&^*p* < 0.0001 vs. Cys+Glu. ^@^*p* < 0.05, ^@@^*p* < 0.01, ^@@@^*p* < 0.001, ^@@@@^*p* < 0.0001 vs. Cys+oe-P2RX7. *n* = 5.

To comprehensively investigate the therapeutic potential and underlying mechanism of targeting TGM2 in Gem-R PDAC, we employed the TGM2 inhibitor Cys. Cys significantly reduced the expression of TGM2 and increased the expression of P2RX7 in Gem-R PDAC cells. Compared to the Cys control group, TGM2 expression was significantly elevated in both Cys+Glu and Cys+oe-P2RX7 treatment groups. Notably, P2RX7 expression remained unchanged in the Cys+Glu group but showed marked upregulation in the Cys+oe-P2RX7 group. When comparing combination treatments, TGM2 expression was further increased in the Cys+Glu+oe-P2RX7 group compared to both Cys+Glu and Cys+oe-P2RX7 monotherapy groups. For P2RX7, the triple treatment group exhibited higher expression than Cys+Glu alone but showed no significant difference compared to the Cys+oe-P2RX7 group (Fig. 9A). This suggests a bidirectional regulatory mechanism between TGM2 and P2RX7, both of which are influenced by Glu. In line with previous reports, TGM2 may activate P2RX7 by facilitating Glu metabolism, while P2RX7 also mediates ATP-promoted TGM2 secretion. As illustrated in Figs. 9B–F and S3C, Cys significantly inhibited the proliferation, migration, and invasion of Gem-R PDAC cells, reduced cellular viability, and promoted apoptosis. Furthermore, TEM analysis revealed that Cys decreased the formation of autophagosomes in Gem-R PDAC cells (Fig. 9G). Western blot results demonstrated that Cys reduced the levels of mitophagy markers parkin and LC3 Ⅱ/Ⅰ, and increased the levels of p62 (Fig. 9H). Treatment with Glu and oe-P2RX7 partially reversed these effects of Cys, with oe-P2RX7 further enhancing the action of Glu. Additionally, Cys decreased the expression of GLS and lowered the levels of ATP, NH_4_^+^, glucose, lactate, glutamate, and extracellular TGM2. However, these effects were abolished following treatment with Glu and oe-P2RX7 (Fig. 9H–L). Meanwhile, similar treatments were conducted in animal experiments. Compared with the Control group, tumor growth in nude mice was significantly inhibited in the Cys group (Fig. 10A, B). Furthermore, Cys downregulated the expression of mitophagy-related proteins parkin and LC3Ⅱ/Ⅰ, and upregulated the expression of p62 in mouse tumor tissues (Fig. 10C). In assessments of Glu metabolism-related indicators, Cys also inhibited GLS expression and reduced the levels of ATP, NH_4_^+^, glucose, lactate, glutamate, and extracellular TGM2 (Fig. 10C–G). Similarly, in the animal model, treatment with Glu and oe-P2RX7 partially reversed the effects of Cys. Moreover, the combined action of Glu and oe-P2RX7 further enhanced this influence. In conclusion, the TGM2-P2RX7 loop regulates Glu metabolism and mitophagy, thereby promoting Gem-R in Gem-R PDAC cells.

### Mitophagy is required for TGM2-P2RX7-driven metabolic reprogramming

**Fig. 11:**
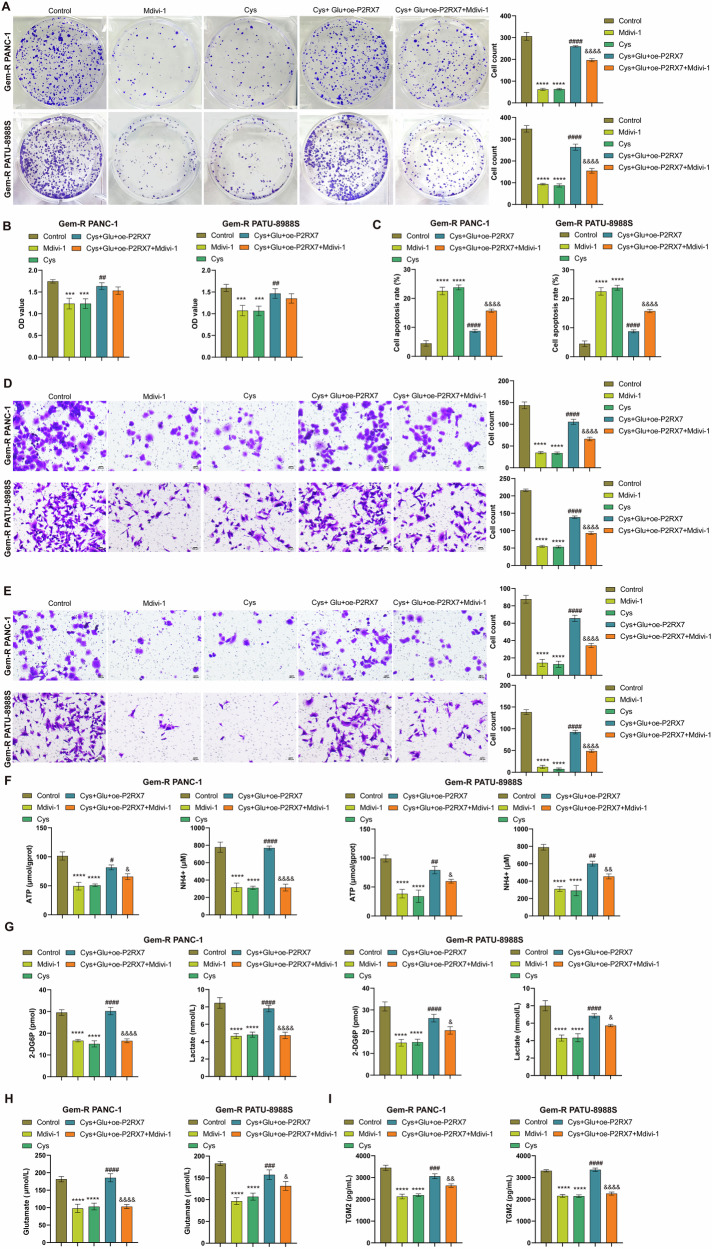
Mitophagy is required for TGM2-P2RX7-driven metabolic reprogramming. **A** Clone formation assay to detect cell proliferation. Scale bar: 100 μm. **B** CCK-8 assay to detect cell viability. **C** Flow cytometry to detect cell apoptosis. **D**, **E** Transwell assay to detect cell invasion and migration. Scale bar: 100 μm. **F** The levels of ATP and NH_4_^+^ were detected in Gem-R PDAC cells. **G** The levels of 2-DG6P and lactate were detected in Gem-R PDAC cells. **H** The level of glutamate was detected in Gem-R PDAC cells. **I** The levels of TGM2 in the supernatants of Gem-R PDAC cells. ^***^p < 0.001, ^****^p < 0.0001 vs. Control. ^##^p < 0.01, ^###^*p* < 0.001, ^####^*p* < 0.0001 vs. Cys. ^&^*p* < 0.05, ^&&^*p* < 0.01, ^&&&&^*p* < 0.0001 vs. Cys+Glu+oe-P2RX7. *n* = 3.

Further, we employed the mitophagy inhibitor Mdivi-1 to validate the necessity of mitophagy in TGM2-P2RX7 loop-driven metabolic reprogramming. Similar to Cys, Mdivi-1 significantly inhibited the proliferation, migration, and invasion of Gem-R PDAC cells, reduced cell viability, and promoted apoptosis. Moreover, it partially abrogated the reversal effects of Glu and oe-P2RX7 on the aforementioned Cys-induced changes (Figs. 11A–E and S3D). Additionally, Mdivi-1 decreased the levels of ATP, NH₄⁺, glucose, lactate, glutamate, and extracellular TGM2. Compared to the Cys +Glu +oe-P2RX7 group, the Cys+Glu +oe-P2RX7+Mdivi-1 group exhibited reduced levels of ATP, NH₄⁺, glucose, lactate, glutamate, and extracellular TGM2 (Fig. 11F–I). Collectively, these results demonstrate that TGM2-P2RX7 loop-driven metabolic reprogramming requires mitophagy.

## Discussion

Insensitivity to chemotherapy is recognized as a primary factor contributing to unfavorable outcomes and increased mortality rates in PDAC. Through our investigation, we have identified TGM2 as a novel oncogenic protein in PDAC. Our findings revealed elevated levels of TGM2 expression in TT tissues compared to TP tissues, with high TGM2 expression correlating with a poor prognosis. Moreover, the expression levels of TGM2 in Gem-R PDAC cells were higher in comparison to normal PDAC cells. TGM2 has the roles of promoting cell proliferation, inhibiting apoptosis, and promoting tumor formation. Mechanistically, TGM2 can regulate Glu metabolism and mitophagy, thereby promoting chemotherapy resistance in PDAC cells. Herein, P2RX7 plays a pivotal role. Thus, our data suggest that the TGM2-P2RX7 loop may represent a promising therapeutic target in PDAC.

The metabolic reprogramming involved in drug resistance in PDAC is also closely associated with chemotherapy, radiotherapy, and immunotherapy, leading to poor prognosis [20]. Glu, being the most prevalent non-essential amino acid in tumor circulation, serves as a significant carbon and nitrogen supplier. Consequently, the robust metabolic utilization of Glu is a prominent characteristic of PDAC both in vivo and in vitro, playing a crucial role in upholding redox balance [21]. Follow-up studies have revealed that Glu is not only involved in tumor growth metabolism, but the activation of its metabolic pathway also promotes tumor drug resistance [22]. Studies have shown that LAT2 can influence mTOR activation by modulating Glu metabolism, thereby decreasing the sensitivity of gemcitabine in PDAC [23]. In this study, we observed significantly elevated levels of Glu metabolites NH_4_^+^ and ATP in cancer tissues from Gem-R PDAC patients compared to adjacent tissues. Concurrently, the addition of exogenous Glu notably increased the expression of GLS and the content of Glu metabolites, thereby promoting the proliferation, invasion, and migration abilities of Gem-R PDAC cells while inhibiting their apoptosis. In vivo experiments further demonstrated that intraperitoneal injection of Glu exhibited a tumor-promoting effect. These findings underscore the crucial role of Glu metabolism in promoting PDAC.

Proteomic analysis has identified TGM2 as a promising biomarker and potential therapeutic target for PDAC [24]. Additionally, the flavonoid kaempferol has been shown to induce apoptosis in PDAC cells through a TGM2-mediated Akt/mTOR signaling pathway [25]. Our study revealed significantly elevated expression levels of TGM2 in TT tissues and PDAC cell lines compared to TP tissues and normal pancreatic ductal cells. High TGM2 levels correlated with a poorer prognosis, underscoring its potential utility as a prognostic biomarker in PDAC. In addition, TGM2 has been implicated in chemoresistance in a variety of cancers. For instance, TGM2 has been linked to chemoresistance in colorectal cancer by activating the Wnt/β-catenin signaling pathway [26]. Furthermore, TGM2 expression has been associated with chemoresistance to gemcitabine in PDAC [10]. Our study confirmed this observation, showing that TGM2 expression was markedly higher in Gem-R PDAC cells compared to normal PDAC cells. Silencing TGM2 markedly inhibited the proliferation, invasion, and migration abilities of Gem-R PDAC cells while promoting their apoptosis. Intriguingly, this effect of TGM2 may be linked to its involvement in Glu metabolism. We found that silencing TGM2 reversed the stimulatory effects of exogenous Glu on the proliferation, invasion, and migration of Gem-R PDAC cells and its inhibitory effect on apoptosis, reducing the expression of GLS and the content of Glu metabolites. Overexpressing TGM2 further enhanced the above effects of exogenous Glu. Concurrently, in a nude mouse model of PDAC established using Gem-R PDAC cells, sh-TGM2 exhibited inhibitory effects on tumor growth and modulated the impact of exogenous Glu. These results suggest that TGM2 promotes Gem-R in PDAC by regulating Glu metabolism.

To further elucidate the mechanisms underlying TGM2’s involvement in Glu metabolism, we conducted RNA sequencing combined with analysis using the KEGG and GO databases to explore Glu-related pathways. GO enrichment analysis revealed significant intergroup variations in the “positive regulation of glutamate secretion” (GO:0014049) pathway. Within this pathway, P2RX7 was the gene most significantly affected by sh-TGM2. The interplay between TGM2 and P2RX7 is intricate. It is known that TGM2 can enhance ATP production by modulating mitochondrial function [13]. P2X7R can be activated by high concentrations of extracellular ATP [12]. Meanwhile, extracellular ATP can also promote the secretion of TGM2 from cells, which can be blocked by selective antagonists of P2X7R [11]. Thus, a complex positive feedback loop is formed between TGM2 and P2X7R. Our study corroborates the bidirectional regulatory axis between TGM2 and P2RX7: TGM2 enhances ATP secretion through Glu metabolism promotion, while extracellular ATP activates P2RX7 to stimulate TGM2 secretion further. This creates a self-perpetuating metabolic-secretory loop. However, an unexpected observation emerged: P2RX7 expression showed an upward trend in sh-TGM2 Gem-R PDAC cells, contradicting previous reports. We speculate that since the active secretion of TGM2 is regulated by ATP-activated P2X7R [11], the reduction in Glu intake after TGM2 silencing, coupled with the cancer cells’ energy demands, leads to sustained activation of P2X7R by exogenous ATP, stimulating mitochondrial GLS energy metabolism signals. Additionally, in the Glu group of Gem-R PDAC cells, Glu promotes P2RX7 expression, which is due to accelerated cellular energy metabolism, where sustained ATP activation induces P2RX7 activation, promoting the active secretion of TGM2 and further facilitating Glu metabolism. However, compared to the sh-TGM2 and Glu groups, P2RX7 expression decreased in the sh-TGM2+Glu group. This may be because the reduction in Glu intake caused by sh-TGM2 was compensated by exogenous Glu, alleviating the energy needs of cancer cells and resulting in a moderate decrease in P2RX7 activity.

P2RX7 is an oncogenic gene with potential in PDAC [27]. Furthermore, extracellular ATP, via P2RX7 signaling, enhances the aggressiveness of vemurafenib-resistant melanoma cells [28]. Herein, we demonstrate that P2RX7 is highly expressed in tumor tissues from Gem-R PDAC patients and promotes the proliferation and aggressiveness of Gem-resistant PDAC cells. Importantly, for the first time, we propose that P2RX7 is involved in Glu metabolism in Gem-PDAC cells. Our results indicate that overexpression of P2RX7 increases the expression of GLS and the content of Glu metabolites. This effect is mediated, on the one hand, by P2RX7-induced secretion of TGM2 in response to extracellular ATP stimulation, indirectly promoting Glu metabolism. On the other hand, it is mediated by the mitophagy-promoting role of P2RX7.

Mitophagy, a crucial homeostatic mechanism for cellular self-degradation, is highly conserved throughout evolution and has recently been implicated in the pathophysiology of tumorigenesis and resistance to anticancer therapies [29–31]. Blocking mitophagy with chloroquine can significantly enhance the sensitivity of PDAC cells to Gem [32]. Intriguingly, the metabolic reprogramming involved in PDAC drug resistance is also closely intertwined with mitophagy. Mitochondrial Glu metabolism plays a pivotal role in maintaining mitochondrial function and biosynthesis in PDAC by replenishing the mitochondrial carbon pool [33]. Correspondingly, mitophagy has also been deemed essential for Glu metabolism in PDAC [34]. Thus, Glu metabolism and mitophagy form a mutually influential loop. Our study also demonstrates that exogenous Glu elevates the level of mitophagy in Gem-PDAC cells. Furthermore, the role of P2RX7 in mitophagy has been established. For instance, soy lectin-triggered IL-6 secretion induces mitophagy to kill intracellular mycobacteria through P2RX7-dependent activation of the JAK2/STAT3/Mcl-1 pathway [35]. P2RX plays a significant role in epilepsy-related mitophagy regulation by fine-tuning HSPB1 expression [36]. Herein, we also found that overexpressing P2RX7 increases the level of mitophagy in Gem-PDAC cells and promotes Glu metabolism. The latter is largely mediated by the role of P2RX7 in mitophagy.

In addition, the correlation between TGM2 and mitophagy has been previously investigated. Studies have demonstrated that inhibiting mitophagy in TGM2-overexpressing cells can reduce cell proliferation and decrease TGM2-induced chemotherapy resistance, revealing a novel role of TGM2-mediated mitophagy in tumor drug resistance [37]. Some reports have elucidated the underlying mechanisms. For instance, TGM2 regulates autophagic function by interacting with LC3B, contributing to enhanced radioresistance in non-small cell lung cancer stem-like cells [38]. However, other studies have shown that inhibiting TGM2 promotes increased Beclin 1-mediated mitophagy, leading to PDAC cell death [39]. In our study, the use of the TGM2 inhibitor Cys significantly decreased the level of mitophagy in Gem-R PDAC cells. Notably, the inhibitory effect of Cys on mitophagy was influenced by oe-P2RX7 and exogenous Glu. This suggests that the promotional effect of TGM2 on mitophagy in Gem-PDAC cells may be mediated by P2RX7 and Glu metabolism. However, the critical role of mitophagy in Glu metabolism requires further experimental validation. This area will constitute a key focus of our future research.

In the intricate regulatory network, the TGM2-P2RX7 axis likely engages in crosstalk with other signaling pathways governing Glu metabolism and mitophagy, such as the mTOR and AMPK pathways—key metabolic regulators [40, 41]. Previous studies demonstrated that extracellular ATP-activated P2RX7 triggers Ca²⁺ influx [42], which subsequently activates AMPK through calcium/calmodulin-dependent protein kinase kinase β (CaMKKβ) [43]. Activated AMPK phosphorylates TSC2, inhibiting mTORC1 and promoting catabolic processes, including mitophagy [44]. This represents a plausible mechanism by which P2RX7 activation modulates mitophagy. Additionally, mTORC1 drives anabolic processes such as protein synthesis [45], while AMPK promotes mitochondrial biogenesis and fatty acid oxidation [46]. The TGM2-P2RX7 axis may balance these pathways by modulating ATP/NH₄⁺ levels, thereby influencing mTORC1-dependent growth signals and AMPK-driven stress responses. In Gem-R PDAC, TGM2-P2RX7-driven metabolic reprogramming likely suppresses AMPK activity through sustained high ATP levels, thereby relieving mTORC1 inhibition. The activated mTORC1 pathway then reciprocally enhances Glu metabolism [47], creating a positive feedback loop that sustains Gem-R. This intricate crosstalk between the TGM2-P2RX7 axis and AMPK-mTOR signaling warrants rigorous investigation in future studies. Collectively, preclinical studies (both in vitro and in vivo) demonstrate that the TGM2-P2RX7 loop plays a pivotal role in modulating glutamate metabolism, mitophagy, and Gem-R in PDAC. Targeting this axis represents a promising therapeutic strategy to improve survival outcomes in patients with Gem-R PDAC. Concurrently, measuring extracellular ATP/TGM2 levels in patient sera may serve as a non-invasive biomarker platform for monitoring chemotherapy resistance. This hypothesis warrants rigorous validation through expanded preclinical studies and clinical trials.

## Materials and methods

### Clinic samples

Clinical data and pathological specimens of tumor tissue (TT) and tumor parasite tissue (TP) of 10 PDAC patients (TNM stages I-II) admitted to Xiangya Hospital, Central South University from February 2024 to July 2024 were collected. Five of them were TT and TP from Gem-R patients with PDAC. After the nursing team assigned groups using a random number table, patients were only told that they were participating in the study, but were not informed of their specific group assignment. Preoperative informed consent was obtained from all participants enrolled in this study, and none of them underwent neoadjuvant therapy. The clinical study was approved by the Ethical Committee of Xiangya Hospital, Central South University (No.202401134). All methods followed the REMARK reporting guidelines. This study exclusively utilized surgical resection specimens of PDAC with adjacent non-cancerous tissues for real-time quantitative PCR (RT-qPCR) and Western blot analyses.

### Cell culture

Human pancreatic duct cells HPNE (iCell-g022, iCell) were cultured with a specialized medium for primary epithelial cells (PriMed-iCell-001, iCell). Human PDAC cells MIA-PACA-2 (CL-0627, Procell) and PANC-1(CL-0184, Procell) were cultured with DMEM medium (11965092, Gbico) with 10% FBS and 1% penicillin/streptomycin (P/S, SV30010, Beyotime). BxPC-3 (iCell-h030, iCell) were cultured with RPMI 1640 medium (11875093, Gbico) with 10% FBS and 1% P/S, PATU-8988S cells (AW-CCH319, Abiowell) were cultured with a specialized medium for PATU-8988S cells (AW-MCH319, Abiowell). The above cells were cultured in a 5% CO_2_ incubator at 37 °C, identified by STR, and were mycoplasma-free.

### Cell treatment

Gem-R PDAC cell lines (Gem-R PANC-1 and Gem-R PATU-8988S) were established through prolonged in vitro exposure to incrementally increasing concentrations of gemcitabine (1, 5, 10, 20, 50, 100, and 200 nM) over a 3-month period [48]. These resistant cell lines were subsequently maintained in culture medium supplemented with 1 µM gemcitabine to preserve the resistant phenotype.

To investigate the effect of TGM2 on Glu metabolism in Gem-R PADC cells, we constructed the following four groups: sh-NC, sh-TGM2, oe-NC, and oe-TGM2. Briefly, Gem-R PANC-1 and Gem-R PATU-8988S cells were transfected with sh-NC, sh-TGM2, oe-NC, or oe-TGM2.

To investigate the role of Glu metabolism in mediating the impact of TGM2 on Gem-R PDAC cells, we grouped the cells as follows: sh-NC, sh-TGM2, Glu, and sh-TGM2+Glu. Specifically, Gem-R PANC-1 and Gem-R PATU-8988S cells transfected with either sh-NC or sh-TGM2 were subjected to treatment with or without Glu (0.1 μM, G7513, Sigma) for a duration of 24 h [49].

To investigate the role of Glu metabolism in mediating the impact of P2RX7 on Gem-R PDAC cells, we grouped the cells as follows: oe-NC, oe-P2RX7, oe-NC+Glu, and oe-P2RX7+Glu. Briefly, Gem-R PANC-1 and Gem-R PATU-8988S cells transfected with oe-NC or oe-P2RX7 were treated with or without Glu for 24 h.

To explore the critical role of ATP in the TGM2-P2RX7 loop, we grouped the cells as follows: Group 1: sh-NC, sh-P2RX7, Glu, Glu+sh-P2RX7, Glu+ATP, and Glu+ATP+sh-P2RX7. Briefly, Gem-R PANC-1 and Gem-R PATU-8988S cells transfected with sh-NC or sh-P2RX7 were treated with Glu or ATP (50 µM) for 24 h [28]. Group 2: sh-NC, Carbenoxolone (CBX), CBX+sh-TGM2, oe-NC, and CBX+oe-TGM2. Briefly, Gem-R PANC-1 and Gem-R PATU-8988S cells transfected with sh-P2RX7 or oe-P2RX7 were treated with CBX (10 µM, HY-B1588, MCE) for 24 h [50].

To explore the mechanism of action between TGM2 and P2RX7, we grouped the cells as follows: NC, oe-TGM2, Glu, Glu+oe-TGM2, and Glu+oe-TGM2+sh-P2RX7. Briefly, Gem-R PANC-1 and Gem-R PATU-8988S cells transfected with oe-TGM2, sh-P2RX7, or their negative control were treated with Glu for 24 h.

To validate the effect of targeting TGM2 on Gem-R PADC cells, we grouped the cells as follows: Control, Cys, Cys+Glu, Cys+oe-P2RX7, and Cys+Glu+oe-P2RX7. Briefly, Gem-R PANC-1 and Gem-R PATU-8988S cells transfected with oe-NC or oe-P2RX7 were treated with Cys (Cystamine, TGM2 inhibitor, 500 μM, C0166, Sigma) [51] or Glu for 24 h.

To explore the necessity of mitophagy in the effect of the TGM2-P2RX7 loop, we grouped the cells as follows: Control, Mdivi-1, Cys, Cys+Glu+oe-P2RX7, and Cys+Glu+oe-P2RX7+Mdivi-1. Briefly, Gem-R PANC-1 and Gem-R PATU-8988S cells transfected with oe-P2RX7 were treated with Cys, Glu, and Mdivi-1 (mitophagy inhibitor, 1 μM, HY-15886, MCE) for 24 h.

### Cell transfection

PANC-1 and PATU-8988S cells (1 × 10^5^ cells) were infected with 20 μL and 100 μL of lentiviruses (1 × 10^8^ TU) for 72 h, respectively. Additionally, 40 μL of HitransG A and 40 μL of HitransG P were added to enhance the infection process. The lentiviruses include sh-NC, sh-TGM2 (HG-SH003551), sh-P2RX7 (HG-SH002562), oe-NC, oe-TGM2 (HG-HO198951), and oe-P2RX7 (HG-HO033951). All of these were purchased from Honorgene. The shRNA sequences of this study are shown in Table 2.

**Table 2:** Target sequences of shRNAs.

Name	Sequences (5′-3′)
sh-NC	TTGACCAGGAGGAGGAGGGAC
sh-TGM2#1	GCCCGTTTTCCACTAAGAGAT
sh-TGM2#2	GGCCAAGTTCATCAAGAACAT
sh-TGM2#3	GGCTGAAGATCAGCACTAAGA
sh-P2RX7#1	ATCTTTTCCTACGTTTGCTTT
sh-P2RX7#2	GTGATGACAAACTTTCTCAAA
sh-P2RX7#3	GGCAATTCAGGGCGGAATAAT

### RT-qPCR

Total RNA was isolated from specimens using the Trizol reagent (Invitrogen). Complementary DNA (cDNA) synthesis was performed through reverse transcription reactions employing the CW2569 mRNA Reverse Transcription Kit (CWBIO). Quantitative real-time PCR assays were executed on the ABI 7900 Real-Time PCR System with fluorescence detection using the Ultra SYBR Mixture (CW2601, CWBIO). Normalization of target gene expression was achieved through comparison with the endogenous control gene β-actin. Quantification analysis followed the 2^-ΔΔCt^ algorithm for relative expression calculation. Detailed primer sequences utilized in this protocol are listed in Table 3.

**Table 3:** Primer sequences for RT-qPCR.

Genes	Sequences (5′-3′)
β-actin_Fwd	ACCCTGAAGTACCCCATCGAG
β-actin_Rev	AGCACAGCCTGGATAGCAAC
TGM2_Fwd	AGTTCCAGTTCGTGCCATC
TGM2_Rev	CACAGACCCATCGTCCTGC
P2RX7_Fwd	ACAAGCTGTACCAGCGGAAA
P2RX7_Rev	GGTGTAGTCTGCGGTGTCAA
AVPR1A_Fwd	TGGGCGCCTTTCTTCATCAT
AVPR1A_Rev	AGGGTTTTCCGATTCGGTCC
ADORA2A_Fwd	GACTGTGACATGGAGCAGGAG
ADORA2A_Rev	ACACGAGGCTTCACCTGAC
KMO_Fwd	CATTGGTGGTGGCTTGGTTG
KMO_Rev	CACGTGTGAAGGTAGCCACT

### Western blot

Cells and tissue samples were lysed with RIPA buffer (AWB0136, Abiowell) to extract total protein, which was then quantified using a BCA kit (AWB0104, Abiowell). Subsequently, the total protein was separated by SDS-PAGE and transferred onto nitrocellulose membranes via gel electrophoresis. The membranes were then incubated overnight at 4 °C with primary antibodies. Afterward, the membranes were immersed in an HRP-linked secondary antibody solution, and detection and visualization were achieved through the application of an ECL chemiluminescence reagent. β-actin acted as the internal standard for normalization. Protein isolation was performed by treating cellular/tissue specimens with RIPA lysis buffer (AWB0136, Abiowell), followed by determination of protein concentration using the BCA assay system (AWB0104, Abiowell). Samples underwent denaturing polyacrylamide gel electrophoresis (SDS-PAGE) for molecular weight-based separation, after which proteins were electrotransferred to nitrocellulose substrates. Membranes underwent prolonged incubation with species-specific primary antibodies at 4 °C. Secondary antibody conjugates bearing horseradish peroxidase (HRP) moieties were applied for immunoreactive signal amplification, with chemiluminescent detection accomplished through ECL substrate incubation. Quantitative normalization employed β-actin expression as the endogenous loading control. The primary antibodies are shown in Table 4. All full and uncropped Western blots are uploaded as “Supplemental Material”.

**Table 4:** Primary antibodies for Western blot.

Name	Code	Dilution ratio	Source
TGM2	15100-1-AP	1:20,000	proteintech
GLS	ab156876	1:5000	abcam
P2RX7	28207-1-AP	1:1500	proteintech
AVPR1A	ab124907	1:5000	abcam
ADORA2A	ab79714	1:1000	abcam
KMO	60029-1-Ig	1:10,000	proteintech
parkin	14060-1-AP	1:2000	proteintech
LC3	14600-1-AP	1:2000	proteintech
p62	18420-1-AP	1:10,000	proteintech
β-actin	66009-1-Ig	1:5000	proteintech

### Determination of the levels of ATP, NH_4_^+^, Glucose uptake, lactic acid (LD), glutamate, and TGM2

In accordance with the manufacturer’s instructions, we utilized the ATP Assay Kit (A095-1-1, Nanjing Jiancheng Bioengineering Institute), the EnzyChromTM Ammonia/Ammonium Assay Kit (ENH3-100, BioAssay Systems), the Glucose Uptake Colorimetric Assay Kit (MAK083, Sigma), the Lactate Assay Kit (A019-2-1, Nanjing Jiancheng Bioengineering Institute), the Glutamate Assay Kit (A074-1-1, Nanjing Jiancheng Bioengineering Institute), and the TGM2 ELISA Kit (CSB-E11797h, Cusabio) to detect the levels of ATP, NH_4_^+^, glucose uptake, lactate, glutamate, and TGM2, respectively.

### Clone formation assay

The cells were digested using 0.25% trypsin (SV30010, Beyotime) and gently rotated to achieve even cell dispersion. Following digestion, the cells were stained with 1 mL of junction staining solution at room temperature for 30 min. After staining, the solution was gently rinsed off with running water, and the cells were left to air dry.

### Cell counting kit 8 (CCK-8) assay

Cellular suspensions were inoculated into 96-well microplates at a density of 5 × 10³ cells per well in 100 μL culture medium. CCK-8 reagent (10 μL/well; Cat# NU679, Dojindo Laboratories) was subsequently introduced to each well. Plates underwent metabolic reduction under humidified 37 °C conditions for 4 h. Spectrophotometric quantification of viable cells was performed at 450 nm using a microplate photometer (MB-530, HEALES).

### Detection of cell apoptosis

Programmed cell death was quantified using the Annexin V-Allophycocyanin (APC) Apoptosis Detection Kit (Cat# KGA1105, KeyGEN BioTECH). Adherent MC3T3-E1 cultures were enzymatically dissociated via trypsinization, followed by centrifugation at 2000 rpm for 5 min. Cell pellets were gently resuspended in 500 μL of proprietary Binding Buffer. The cellular suspension received 5 μL aliquots of both fluorochrome-conjugated Annexin V-APC and nuclear stain Propidium Iodide (PI), with subsequent dark incubation for 10 min at ambient temperature. Fluorescence-based cytometric analysis was performed on a dedicated flow cytometry platform to resolve apoptotic populations.

### Transwell assay

In the cell migration assay, the lower chamber of the transwell was filled with 500 μL of Complete Medium containing 10% FBS. Cells were detached using trypsin, suspended in serum-free medium at a concentration of 2 × 10^6^ cells/mL, and 100 μL of the cell suspension was added to each upper chamber well. The cells were allowed to migrate for 48 h at 37 °C. Following incubation, the upper chamber was gently extracted and transferred to new wells containing PBS. After rinsing the upper chamber thrice with PBS to eliminate any remaining cells, the membrane was fixed in 4% paraformaldehyde for 20 min. Subsequently, the membrane was stained with a 0.1% crystal violet solution for 5 min, washed with water, and examined under a microscope (DSZ2000X, Cnmicro) to visualize and analyze the migrated cells.

For the assessment of cell invasion ability, in addition to preparing the sterile gun tip, EP tube, Matrigel, and Transwell, it was essential to pre-cool the items overnight at 4 °C one day in advance. The cells were then mixed with Matrigel to a final concentration of 200 μg per well and incubated for 30 min at 37 °C. Subsequently, the process mirrors that of the migration assay described above.

### Animal and treatment

Four-week-old female BALB/c nude mice were obtained from Hunan Slake Jingda Experimental Animal Co., Ltd. The mice were randomly allocated to different groups based on the cell grouping criteria, with five mice in each group. One week after the mice were acclimatized, Gem-R PATU8988S and Gem-R PANC-1 cells transfected with sh-NC, sh-TGM2, oe-NC, or oe-P2RX7 were injected subcutaneously into the right axilla. After tumor implantation, tumor measurements were observed twice a week. After 10 days of waiting for tumor implantation, mice were treated with Glu (intraperitoneal injection, 1 g/kg, once a day) [52], or Cys (intraperitoneal injection, 20 mg/kg, once a day) [53]. At the end of the experiment, nude mouse seed tumor tissues were taken for photographing and weighing. The animal study was approved by the Animal Ethics Committee of Xiangya Hospital, Central South University (No.202401172). All methods were performed according to the ARRIVE guidelines.

### Immunohistochemistry (IHC)

Tissue sections were initially deparaffinized through xylene exposure, followed by graded ethanol hydration. Specimens underwent antigen retrieval via 50% uric acid exposure (37 °C, 30 min), then enzymatic digestion with trypsin solution under identical temperature conditions. Endogenous peroxidase activity was neutralized through 1% periodic acid treatment (10 min). Immunohistochemical reactions proceeded with overnight refrigerated incubation (4 °C) using rabbit anti-TGM2 primary antibody (1:200 dilution; Cat# 15100-1-AP, Proteintech). Sequential secondary labeling employed goat anti-rabbit IgG H&L conjugate (Cat# 31460, Invitrogen) at 37 °C for 30 min. Chromogenic visualization utilized DAB substrate (ZLI-9018, Beijing Zhongsui Jinqiao Biotechnology Co.), with nuclear counterstaining achieved through hematoxylin application (AWI0001a, Abiowell).

### RNA sequencing

RNA sequencing was conducted as previously described [54]. Total RNA was extracted utilizing Trizol reagent (Invitrogen) to enrich mRNA. The enriched mRNA was then reverse transcribed to create double-stranded cDNA, followed by purification, library construction, quality assessment, and sequencing. Raw data underwent processing for adapter trimming and filtering of low-quality reads using the fastp software. The filtered data was aligned to the reference genome sequence with HISAT2 to generate the aligned reference genome. Expression level differential analysis was performed using Stringtie software, and enrichment analysis was used to identify relevant pathways (KEGG) and functions (GO).

### Transmission electron microscopy (TEM)

Specimens underwent dual fixation: initial preservation in 2.5% glutaraldehyde followed by secondary stabilization with osmium tetroxide vapor. Ultrathin sectioning (50–70 nm) was executed using an ultramicrotome, with subsequent heavy metal staining via uranyl acetate and lead citrate solutions. Stained grids received triple-distilled water rinses before mounting on copper mesh supports. Morphological evaluation employed a transmission electron microscope (Tecnai G2 Spirit, FEI Company, USA) operating at 80 kV, with imaging conducted across 5000–10,000× magnification ranges. Pathological features, including fragmented mitochondrial cristae and double-membrane autophagic vacuoles, were annotated by certified histopathologists through digital image analysis.

### Dual-luciferase reporter assay

Computational analysis of TGM2 binding motifs within the P2RX7 mRNA 3′ UTR was performed using the JASPAR database (https://jaspar.elixir.no/). 293T cells (HG-NC071, HonorGene) underwent simultaneous transfection with either TGM2 overexpression (oe-TGM2) or knockdown (sh-TGM2) vectors alongside P2RX7 promoter-reporter constructs. All plasmid reagents were sourced from HonorGene. For cell lysis, culture dishes were gently agitated at room temperature for 10 min post treatment. Dual luciferase activity was measured using a GloMax 20/20 luminometer (Promega) following sequential reagent addition: firefly luciferase activity was recorded first, after which the reaction mix was supplemented with stop solution and Renilla luciferase substrate to initiate the secondary reaction. Relative luciferase activity was calculated by normalizing Renilla signals to their corresponding firefly luciferase values.

### Co-immunoprecipitation (Co-IP)

Cell lysates were used to extract proteins with 300 μL IP lysis solution (AWB0144, Abiowell). These proteins were then incubated overnight at 4 °C with either normal rabbit IgG (B900610, Proteintech) or an antibody targeting TGM2 (15100-1-AP, Proteintech). To guarantee precise protein binding, the mixtures were processed with protein A/G agarose beads for a duration of 2 h at 4 °C. Subsequently, the immune complexes underwent heat denaturation at 95 °C for 5 min and were analyzed through Western blot.

### Statistical analysis

Quantitative data analysis was conducted using GraphPad Prism version 8.0.2. All experimental datasets derived from triplicate biological repeats were represented as arithmetic means ± standard deviations. Between-group comparisons for paired samples utilized the two-tailed Student’s *t*-test. Multifactorial experimental designs employed analysis of variance (ANOVA), with one-way ANOVA selected for single-variable comparisons and two-way ANOVA implemented for dual-factor analyses. Statistical significance was defined at the 95% confidence level (*α* = 0.05), with *p*-values < 0.05 considered indicative of meaningful biological differences.

## Supplementary information

Original Western Blot Images-1

Original Western Blot Images-2

Original Western Blot Images-3

Original Western Blot Images-4

Supplementary Materials

## Data Availability

All data can be obtained from the corresponding author Shuai Liang.
