# Supplementary figures and images for: TGM2-P2RX7 loop promotes gemcitabine resistance in pancreatic cancer by modulating glutamine metabolism and mitophagy
Source: Cell Death Discov. 2025 Dec 30;12:2. doi: 10.1038/s41420-025-02922-x (PMC12780038; doi:10.1038/s41420-025-02922-x)

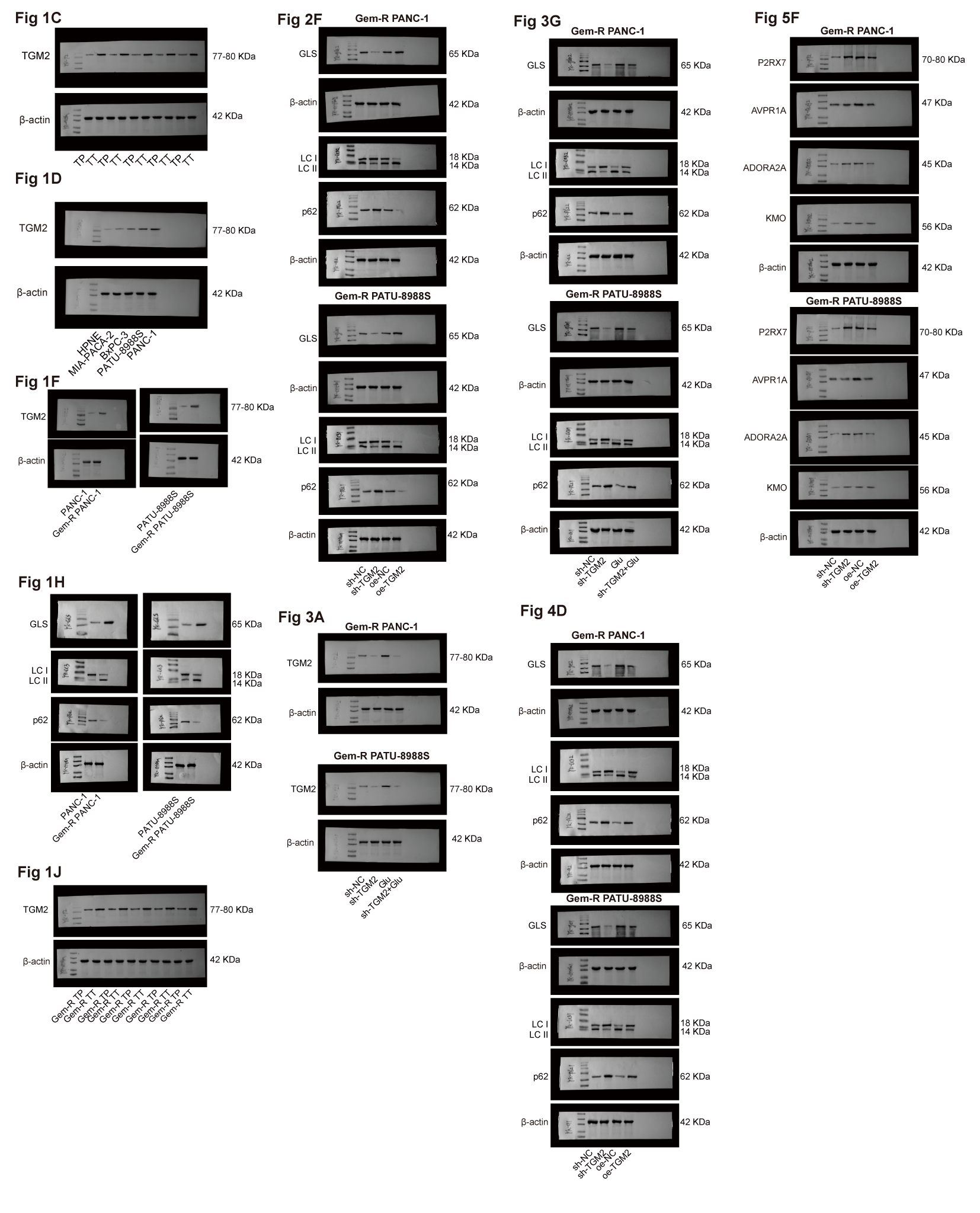

Supplement: Supplementary file 1 — Original Western Blot Images-1 [file 41420_2025_2922_MOESM1_ESM.tif]

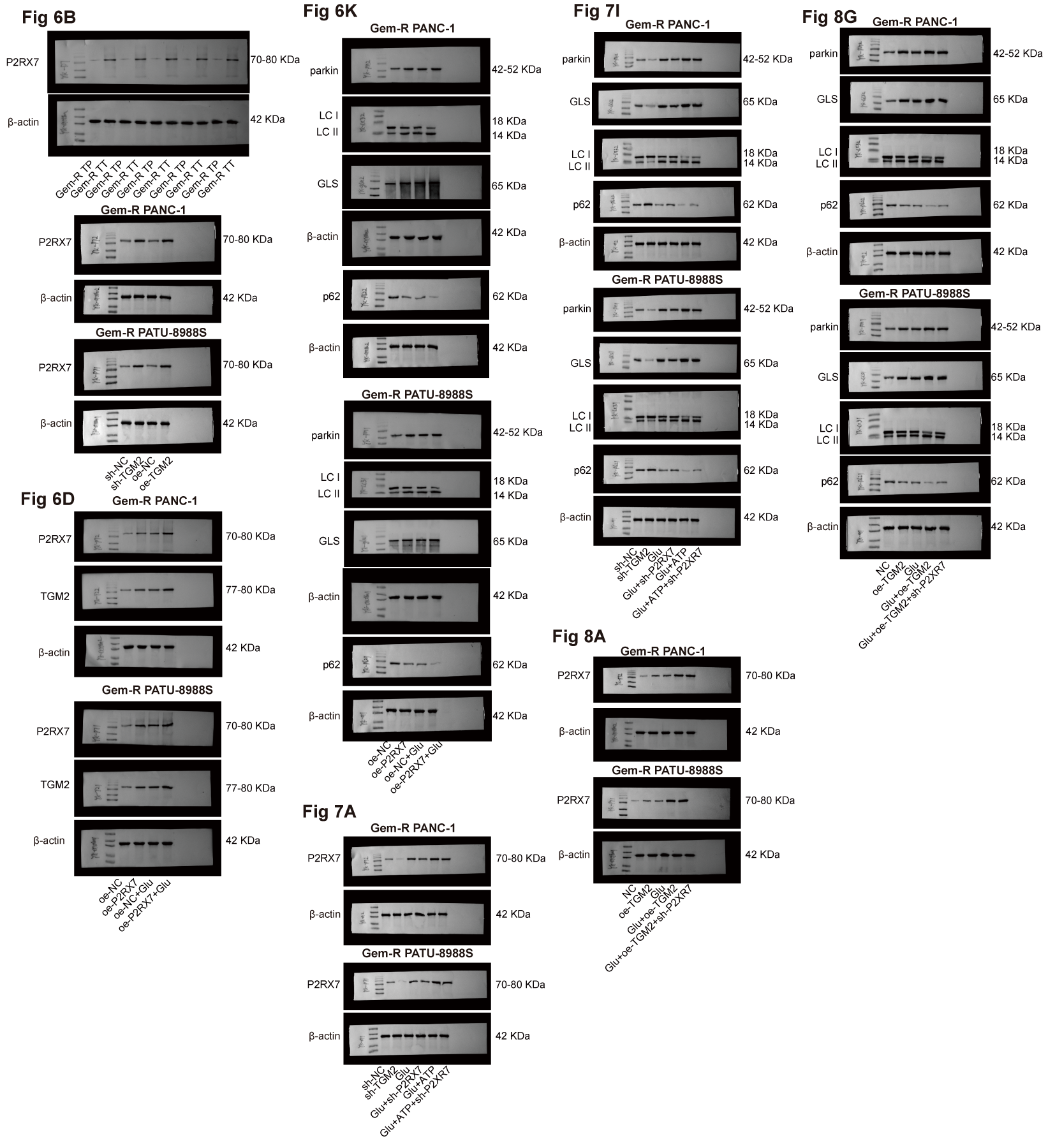

Supplement: Supplementary file 2 — Original Western Blot Images-2 [file 41420_2025_2922_MOESM2_ESM.tif]

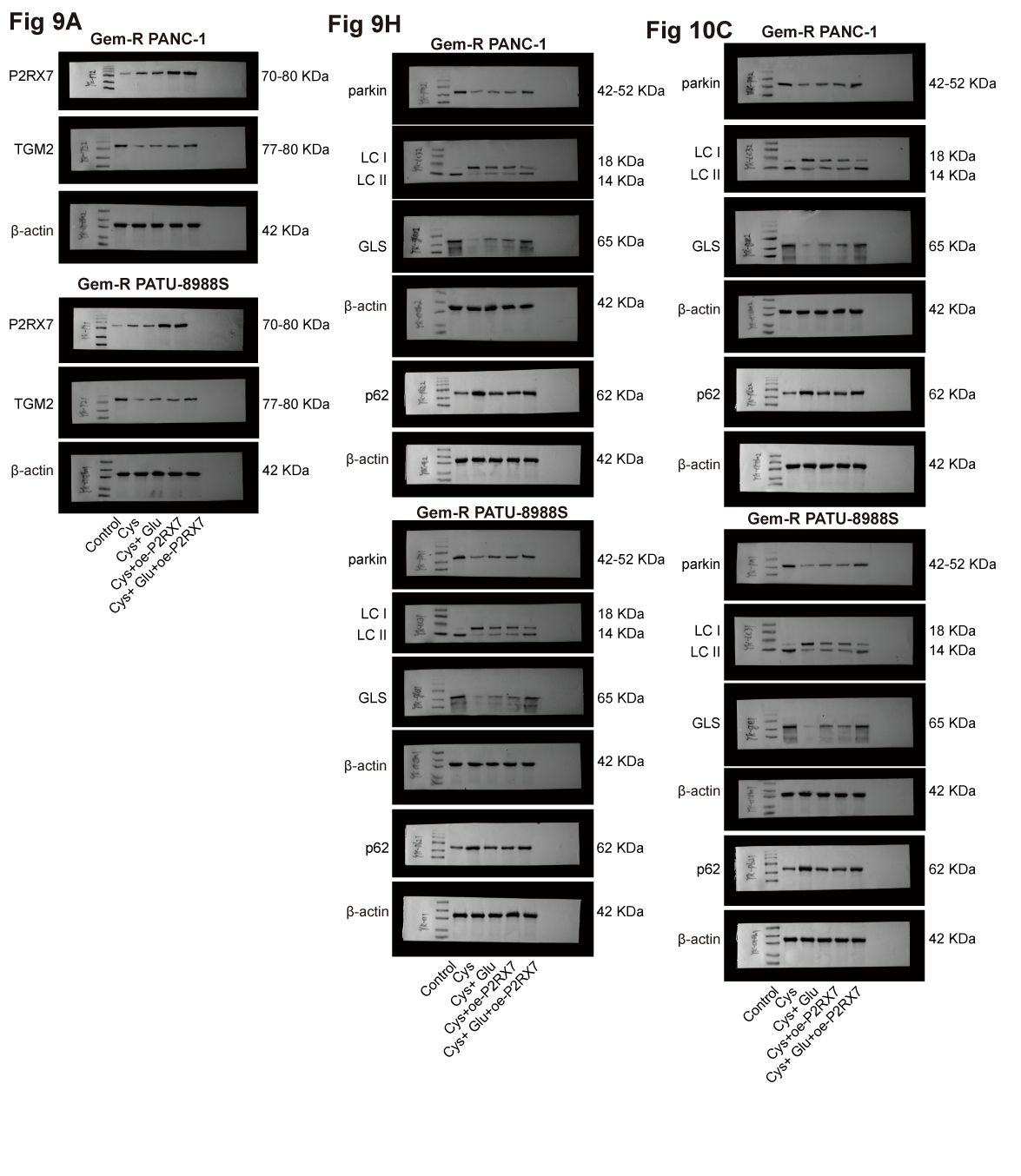

Supplement: Supplementary file 3 — Original Western Blot Images-3 [file 41420_2025_2922_MOESM3_ESM.tif]

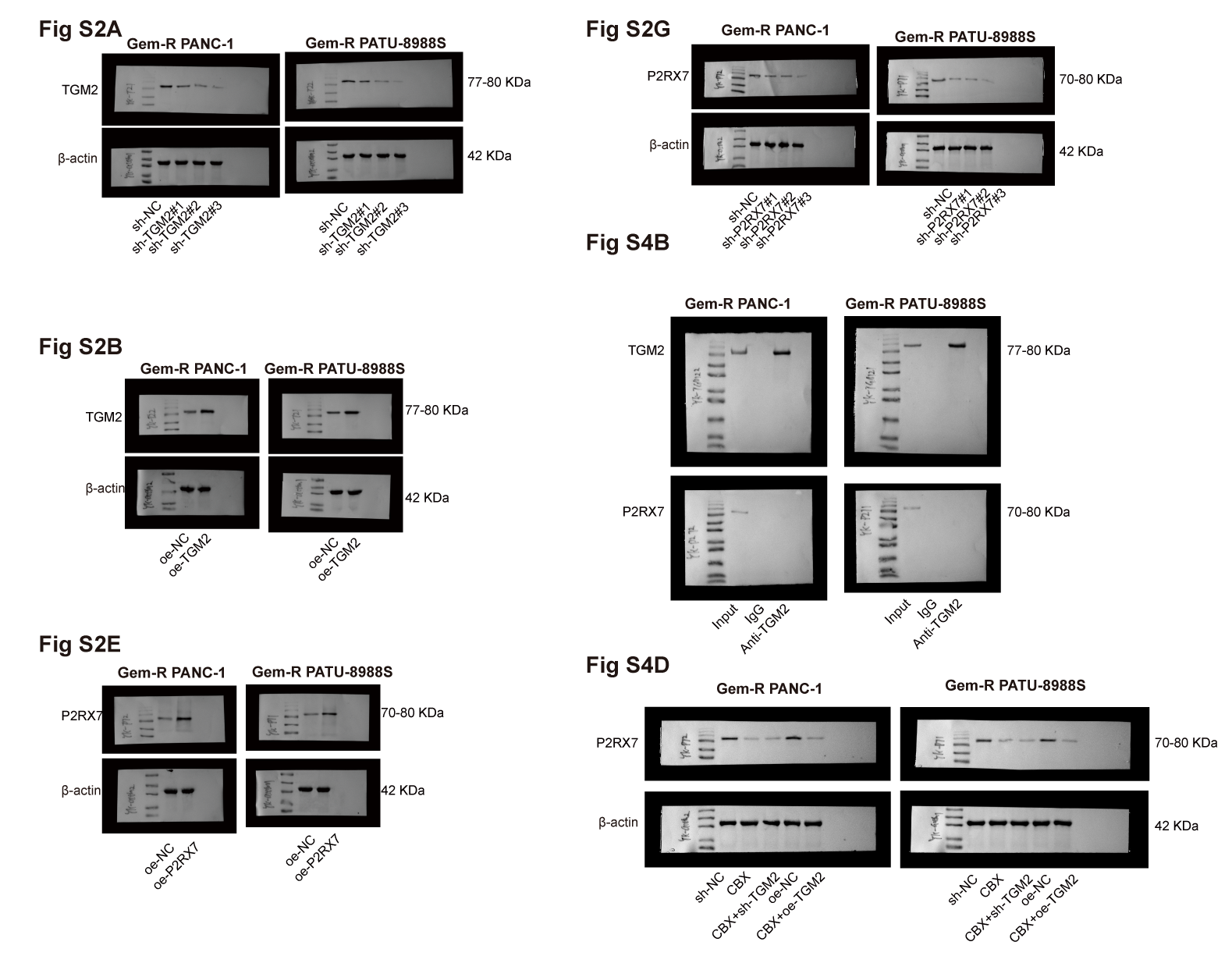

Supplement: Supplementary file 4 — Original Western Blot Images-4 [file 41420_2025_2922_MOESM4_ESM.tif]
