# Supplementary material for: TGM2-P2RX7 loop promotes gemcitabine resistance in pancreatic cancer by modulating glutamine metabolism and mitophagy
Source: Cell Death Discov. 2025 Dec 30;12:2. doi: 10.1038/s41420-025-02922-x (PMC12780038; doi:10.1038/s41420-025-02922-x)
Supplement: Supplementary file 5 — Supplementary Materials [file 41420_2025_2922_MOESM5_ESM.docx]

**Supplementary Materials**

**
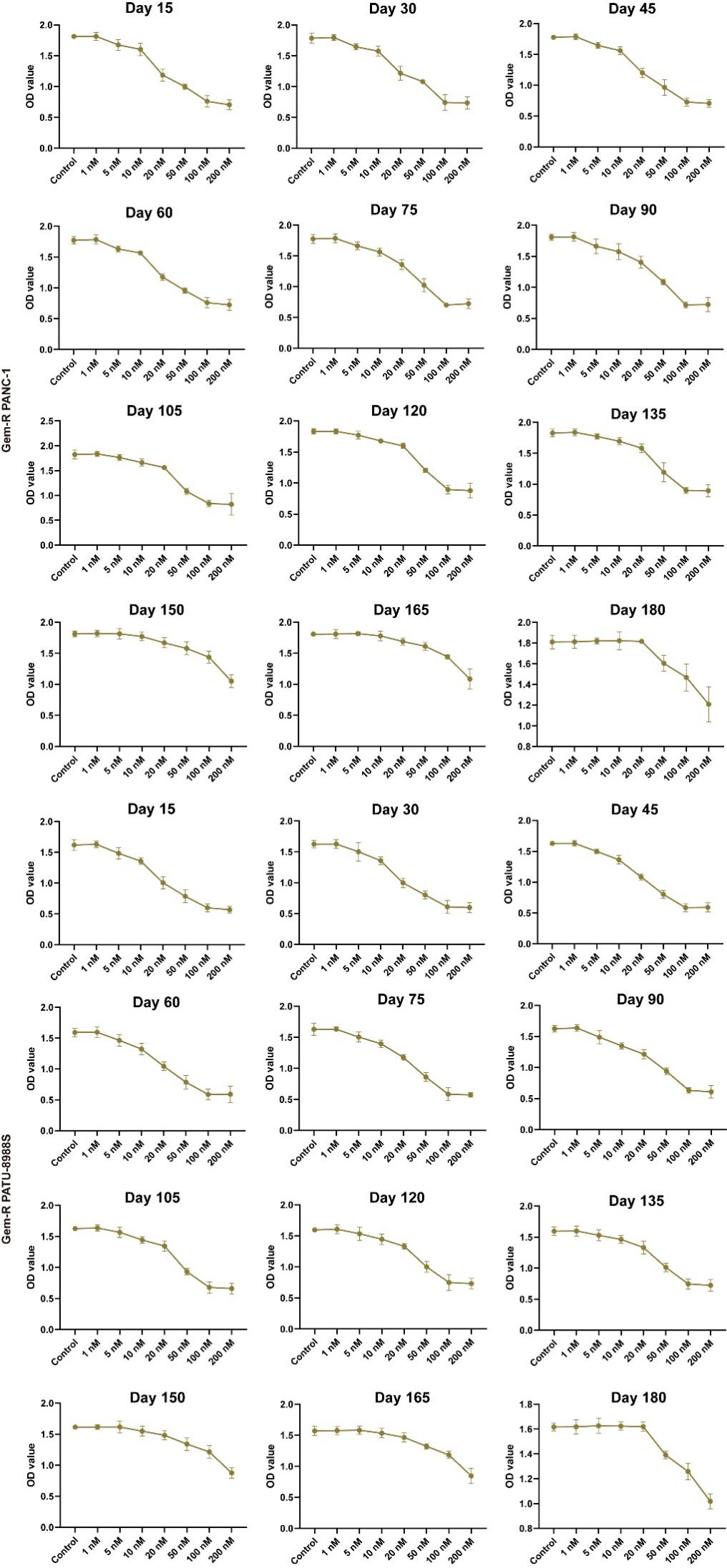
**

**Figure S1.** Construction of Gem-R PDAC cells.

**
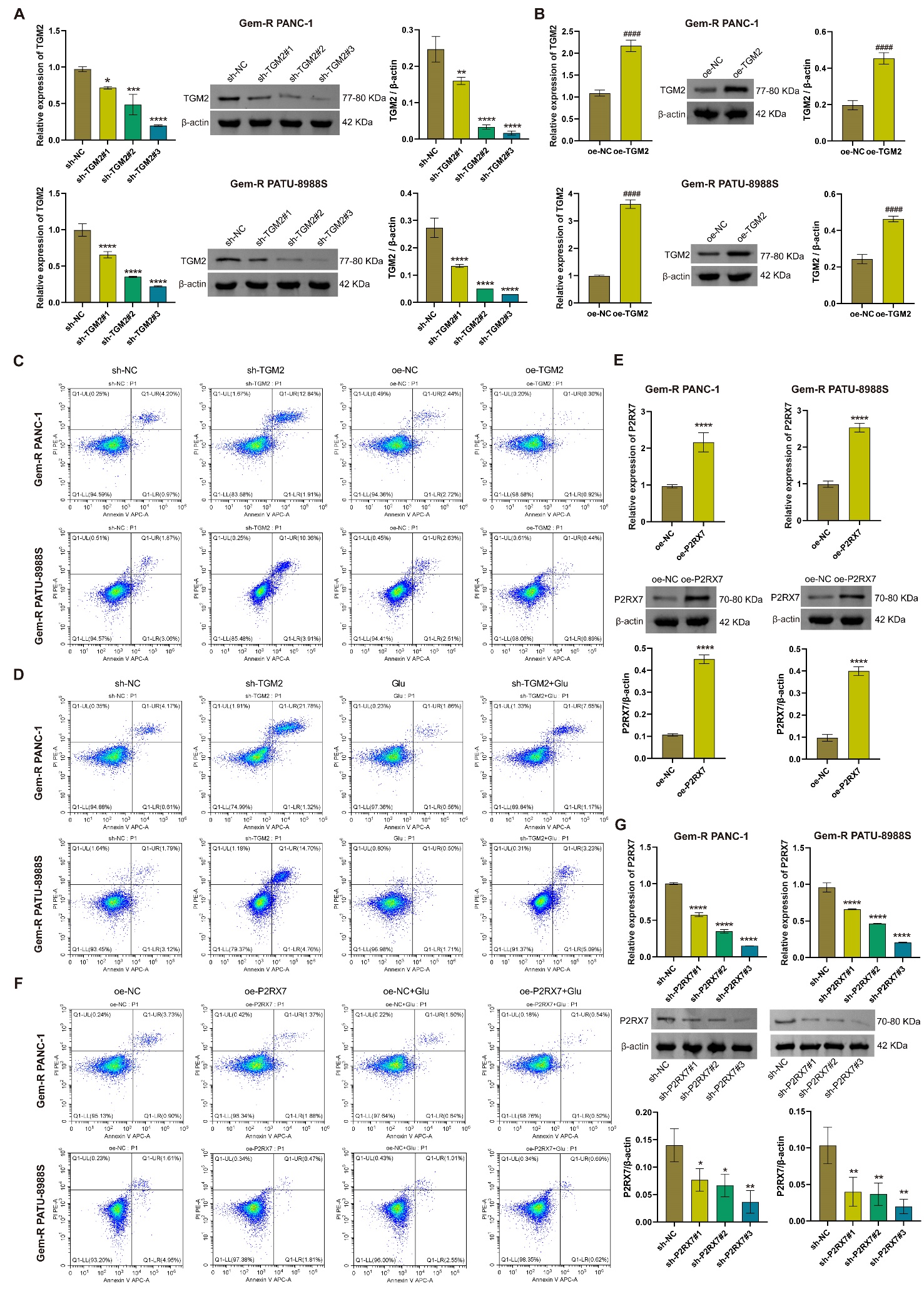
**

**Figure S2.** A. RT-qPCR and Western blot analysis of TGM2 expression in Gem-R PDAC cells transfected with sh-NC or sh-TGM2. *p<0.05, **p<0.01, ***p<0.001, ****p<0.0001 vs. sh-NC. B. RT-qPCR and Western blot analysis of TGM2 expression in Gem-R PDAC cells transfected with oe-NC or oe-TGM2. ####p<0.0001 vs. oe-NC. C-D. Representative images of cell apoptosis by flow cytometry. E. RT-qPCR and Western blot analysis of P2RX7 expression in Gem-R PDAC cells. ****p<0.0001 vs. oe-NC. F. Representative images of cell apoptosis by flow cytometry. G. RT-qPCR and Western blot analysis of P2RX7 expression in Gem-R PDAC cells. *p<0.05, **p<0.01, ****p<0.0001 vs. sh-NC. n=3.


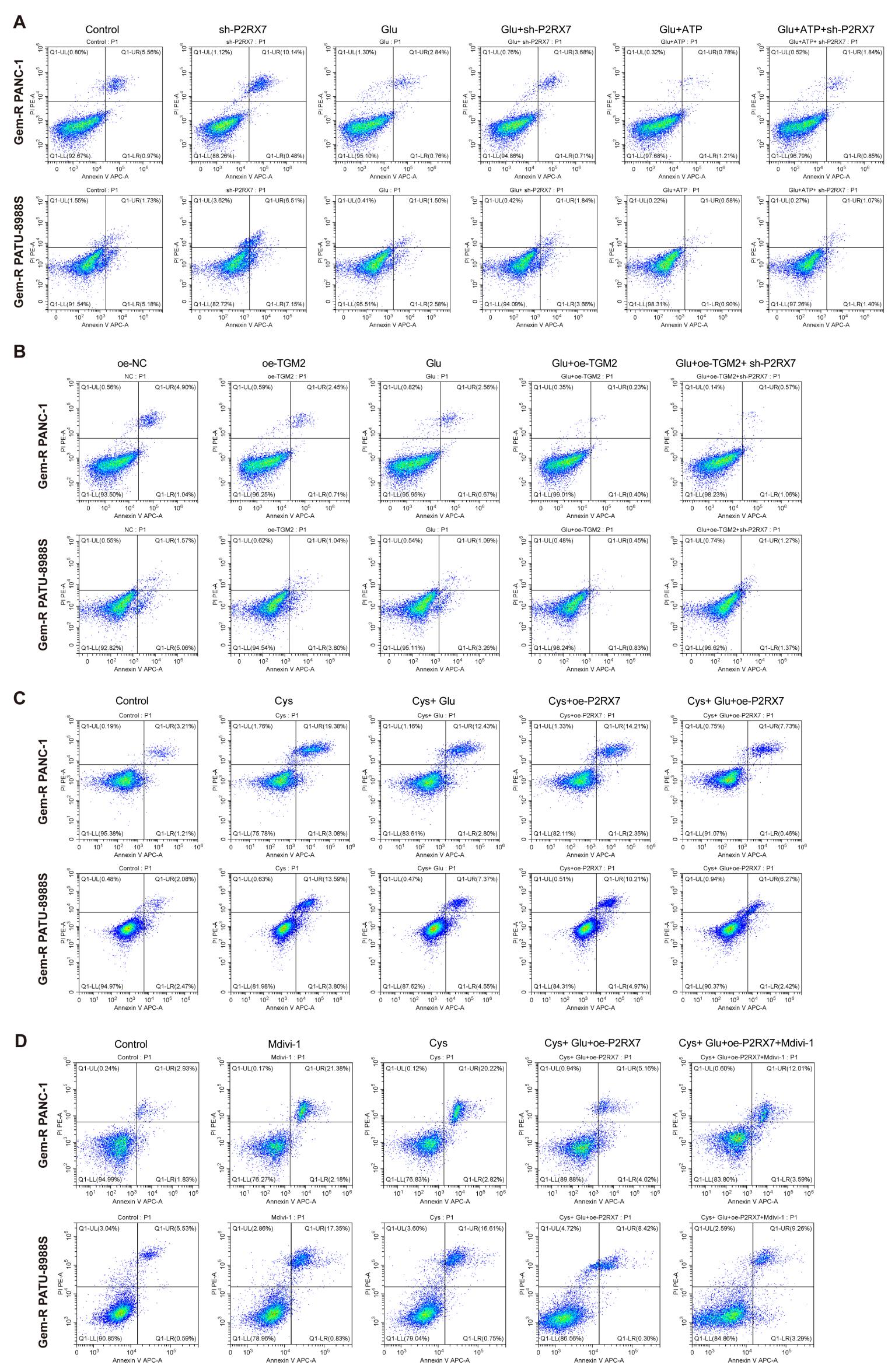


**Figure S3.** A-D. Representative images of cell apoptosis by flow cytometry.


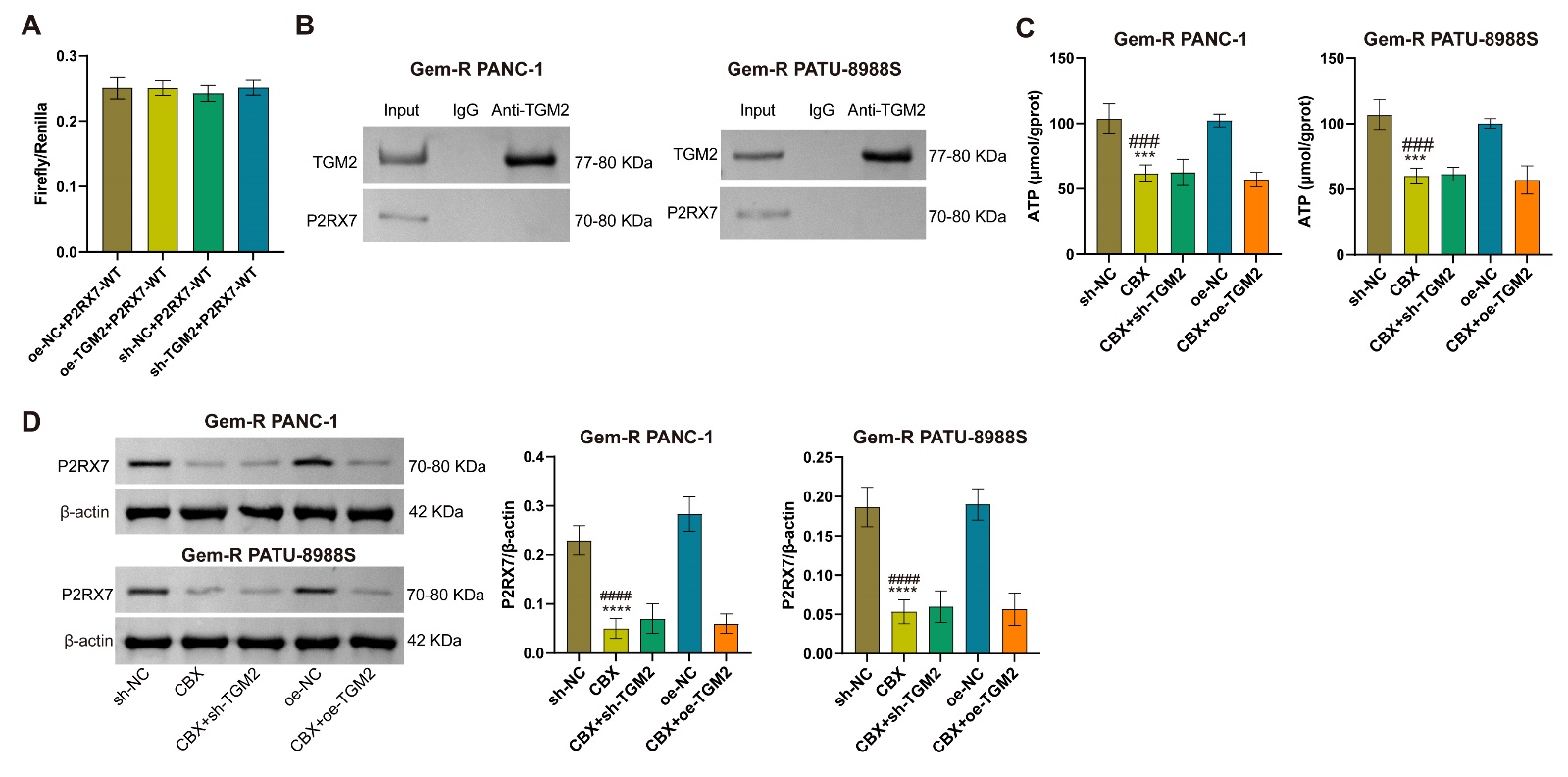


**Figure S4.** A. The dual-luciferase reporter assay was used to verify the transcriptional regulatory relationship between TGM2 and P2RX7. B. Co-IP assay for detecting the interaction of TGM2 with P2RX7. C. The levels of ATP were detected in Gem-R PDAC cells. D. Western blot analysis of P2RX7 expression in Gem-R PDAC cells. ***p<0.001, ****p<0.0001 vs. sh-NC. ###p<0.001, ####p<0.0001 vs. oe-NC. n=3.
